# From Pores to Pavement: Advanced Modeling of Aluminosilicates for Scalable Carbon Capture in Concrete

**DOI:** 10.1002/advs.202517317

**Published:** 2025-11-07

**Authors:** Pooja Anil Kumar Nair, Jéssica de O. N. Ribeiro, Murilo Daniel de Mello Innocentini, Kevin Paine, Juliana Calabria‐Holley

**Affiliations:** ^1^ RENEW Centre for Regenerative Engineering and Design for a Net Positive World Department of Architecture and Civil Engineering University of Bath Claverton Down BA2 7AY UK; ^2^ Normet UK Ltd Coventry CV3 4LA UK; ^3^ Department of Chemical and Materials Engineering Federal University of Lavras Ekaterinburg 37203202 Brazil; ^4^ Postgraduate Programme in Environmental Technology University of Ribeirão Preto Ribeirão Preto 14096‐900 Brazil; ^5^ Centre for Climate Adaptation and Environment Research Department of Architecture and Civil Engineering University of Bath Claverton Down BA2 7AY UK

**Keywords:** carbon capture materials, cementitious composites, engineered aluminosilicates, scalable building materials, universal isotherm modeling

## Abstract

This work marks a significant advance in developing scalable, material‐based solutions for carbon capture. Universal Isotherm Modeling (UIM) provides a transferable modeling approach applicable to a wide range of porous materials, laying a foundation for future innovations in carbon capture. Although focused on innovative construction materials, the impact of this study extends across environmental engineering, materials science, and industrial decarbonisation, with implications for membrane technologies and adsorbent optimisation. Combining UIM with experimental data, the effects of alumina content, solvent choice, and amine functionalization were investigated on CO_2_ uptake in sol‐gel synthesied aluminosilicates. UIM analysis demonstrated a powerful influence of ultramicropores (0.3–0.4 nm), alumina inclusion, and amine grafting. Ultramicropores are crucial in creating high‐energy adsorption sites (S_1_), essential for capturing CO_2_ at low concentrations. Conversely, alumina and amine grafting affect lower‐energy sites (S_2_, S_3_), which activate at higher pressures and boost overall carbon capture capacity. These findings, supported by analytical tools such as gas adsorption measurements, were benchmarked against faujasite, a well‐established reference material. This work introduces a predictive framework linking material structure and chemistry to adsorption energetics, an integration that enables targeted design of advanced carbon capture materials. Replacing guesswork with molecular insight accelerates the discovery of streamlined selective sorbents.

## Graphical abstract

This study presents a transferable modeling framework for carbon capture using aluminosilicates, integrating Universal Isotherm Modeling with experimental data. It reveals how ultramicropores, alumina content, and amine functionalization influence CO_2_ adsorption energetics. The findings offer molecular‐level insights that enable predictive design of advanced sorbents, promising breakthroughs in scalable decarbonization technologies.

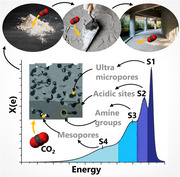

## 1 Introduction

Portland cement (PC) has a huge carbon footprint and contributes significantly to global warming. The production of PC results in emissions of ≈0.8–0.9 tCO_2_/t, with 50–60% from calcium carbonate decomposition and the rest from fuel combustion. PC global production releases 2.3 Gt of CO_2_ annually and accounts for 8–9% of global anthropogenic CO_2_ emissions and 2–3% of energy use.^[^ ^1^ ^]^ As a comparison, the UK cement industry emitted 7.3 Mt of CO_2_ in 2020, equivalent to 1.5% of total UK CO_2_ emissions and 9% of manufacturing emissions.^[^ ^2^ ^]^

Although the cement and concrete industries have proposed pathways to achieve net zero emissions (NZE) and limit global warming to 1.5 °C by 2050, recent evidences indicate that current global policies are unlikely to meet this goal, highlighting an increasingly critical climate situation.^[^ ^3^, ^4^, ^5^ ^]^ The strategies can be broadly classified into two categories: i) reducing carbon emissions^[^ ^3^, ^4^ ^]^ and ii) increasing carbon sequestration.^[^ ^6^, ^7^ ^]^ The former category prioritizes energy efficiency, clinker‐to‐cement ratio reduction, low‐carbon fuel use, etc., whereas the latter focuses on Carbon Capture, Usage, and Storage (CCUS) technologies to maximize and stabilize carbon in building materials.^[^ ^8^ ^]^ These efforts are part of the Global Cement and Concrete Association (GCCA) roadmap^[^ ^9^ ^]^ and aligned with the 2030 UN Sustainable Development Goals, particularly SDG‐9, SDG‐11, and SDG‐13, which aim to build a low‐carbon future.^[^ ^10^ ^]^

During the service life of cement materials, their alkaline phases are attacked by atmospheric CO_2_ and precipitate stable inorganic carbonates, acting as a carbon sink.^[^ ^6^ ^]^ The carbon uptake due to passive carbonation can reach 0.49 tCO_2_/t_cement_ or 25–43% of the clinker production emissions.^[^ ^11^ ^]^ Unfortunately, passive carbonation, unlike rapid CO_2_ emissions during limestone calcination, is a slow and complex process that persists throughout the life cycle of concrete (50–100 years),^[^ ^12^, ^13^ ^]^ making it difficult to offset emissions. Carbonation of PC may eventually reduce concrete cover alkalinity and increase reinforcement corrosion risk, impacting structural strength and safety.^[^ ^14^, ^15^ ^]^

The incorporation of nanomaterial additives and supplementary cementitious materials is a potentially effective CCUS strategy for enhancing CO_2_ sequestration through carbonation in concrete while preserving its strength and durability.^[^ ^3^, ^16^, ^17^ ^]^ Although the use of naturally occurring pozzolans and CO_2_‐binding minerals may provide a more immediate and scalable solution, the depletion of these natural resources may also have a long‐term impact on the environment. On the other hand, zeolites,^[^ ^18^, ^19^, ^20^ ^]^ geopolymers^[^ ^3^ ^]^ and engineered synthetic aluminosilicates (ESA)^[^ ^21^ ^]^ are examples of man‐made materials that could not only yield an optimized environment for hydration and carbonation of cement pastes, but also boost the CO_2_ uptake in concrete.^[^ ^22^ ^]^ Contrary to zeolites, aluminosilicates prepared under mild conditions via the sol‐gel method have an amorphous cross‐scale hierarchical microstructure, making them versatile materials with applications in catalysis, adsorption, membranes, and more.^[^ ^23^, ^24^ ^]^ ESA are especially promising for cement paste incorporation since their chemical composition and molecular structure can be fine‐tuned by the sol‐gel technology and yield particles of controlled pore size distribution, porosity, and surface area. Furthermore, previous research of this group has established unequivocal evidence that the carbonation mechanism within the PC matrix can be enhanced using ESA without affecting the mechanical performance at later ages.^[^ ^21^ ^]^

Understanding the adsorption phenomena is key to tailoring the selectivity, working capacity, and regenerability of ESA by the sol‐gel technique to maximize their CO_2_ uptake. The porous adsorbents provide sites with a favorable energy state to accommodate the adsorbate molecules in mono‐ or multilayer formations. The unique interactions between adsorbate and adsorbent can be experimentally assessed by an adsorption isotherm, the amount of fluid molecules retained onto the pore surfaces of the material at a fixed temperature as a function of pressure.^[^ ^25^, ^26^ ^]^

The universal isotherm model (UIM) was recently developed by Ng et al.^[^ ^27^ ^]^ to overcome the limitations of the empirical and theoretical isotherm models.^[^ ^26^ ^]^ The theory behind the UIM combines three concepts: the Homotattic Patch Approximation (HPA),^[^ ^28^ ^]^ a revised Langmuir model, and the fractional probability factor for the distribution of site energy sets.^[^ ^29^ ^]^

The UIM is able to describe the shapes of all six IUPAC isotherm types and gives the characteristics of the adsorption surface in the form of the distribution of adsorption energy sites and their availability.^[^ ^29^, ^30^ ^]^ The tool is helpful to compute the site energies over heterogeneous surfaces in natural or engineered materials and thus determine the total uptake at different pressure ratios.

Despite being recent, the UIM has been successful in relating the chemical formulation and processing variables of different porous solids and the adsorption process. Ribeiro et al.^[^ ^31^ ^]^ used the UIM to study how engineered properties influenced the adsorption performance of water on zirconium‐containing mesoporous silica. Burhan et al.^[^ ^30^ ^]^ used the UIM to tailor the characteristics of solid desiccants for air dehumidification.

The literature has documented CO_2_ adsorption isotherms for a range of porous solids^[^ ^20^, ^32^, ^33^, ^34^, ^35^, ^36^ ^]^ with the objective of developing CCUS strategies to mitigate climate change. However, limited attention has been given to the development of synthetic engineered materials that could improve the carbon sequestration coefficient of concretes while simultaneously enhancing the hydration and carbonation reactivity in cement pastes, without compromising their mechanical performance or durability.^[^ ^21^ ^]^ More widely, beyond the CCUS strategies, there is also a lack of prior research that has methodically used the UIM to correlate energy sites and pore size distribution, allowing processing variables to be manipulated to increase CO_2_ adsorption in sol‐gel aluminosilicates.

This multidisciplinary project is part of a net‐positive framework that uses soft‐chemistry routes to engineer novel and net‐zero building materials for a sustainable world and an important climate change mitigation technology. In the current study, sol‐gel aluminosilicates were synthesized, and their adsorptive properties were modified by varying the type and proportion of the alumina precursor and the solvent content. Part of the formulations was functionalized by amine grafting to further enhance the CO_2_ adsorption. The resulting microstructures were investigated by Fourier transform infrared spectroscopy, thermogravimetric analysis, energy dispersive spectroscopy, and CO_2_ adsorption. The UIM was used to quantify the groups of adsorption sites according to their energy profile and correlate them with the pore size distributions. The adsorption performance of the produced ESA was validated by comparison with Faujasite as a benchmark material.

By systematically engineering the pore architecture and surface chemistry of ESA sorbents, this work enables a direct translation from molecular‐level optimization to practical incorporation into cementitious composites. The optimized ESA materials can be blended into concrete mixes, with subsequent performance verification confirming both enhanced CO_2_ uptake and compatibility with mechanical requirements. This approach offers a scalable solution for deploying carbon‐capturing additives in high‐volume construction applications, such as pavements, thereby maximizing both environmental benefits and structural functionality in the built environment.

## 2 Experimental Section

The aluminosilicate samples synthesized in this work (**Table** **1**) were developed based on an initial Portland cement‐compatible silicate sample (T‐100). The optimum carbon capture ESA samples were obtained after a three‐stage development. Stage I: alumina to silica ratio optimization; Stage II, engineering optimum pore size, and Stage III, amine grafting as described below and presented in Table 1.

**Table 1:** Variation of the molar ratio of reactants with respect to 1 mol silica.

ESA Series	Sample ID	SiO_2_	Al_2_O_3_	EtOH	H_2_O	NaOH
Series I	T‐100	1	0	10	10	0
	T‐97.5	1	0.025	10	10	0
	T‐96.25	1	0.04	10	10	0
	T‐95	1	0.05	10	10	0
	T‐90	1	0.10	10	10	0
	T‐66.67	1	1	10	10	0
Series II	T‐95‐AlO(OH)	1	0.05	10	10	0
	T‐90‐NS	1	0.10	0	10	0
	T‐90NSNa	1	0.10	0	18	0.462
Series III	T‐100‐NH_2_	1	0	10	10	0
	T‐96.25‐NH_2_	1	0.04	10	10	0

### 2.1 Synthesis and Development of ESA

Aluminosilicates were synthesized using tetraethoxysilane (TEOS, CAS 78‐10‐4, 98% purity, Fisher Scientific) and aluminum nitrate nonahydrate (ANN, CAS 7784‐27‐2, 99.99% purity, Fisher Scientific) as the silica and alumina precursors, respectively. The solvent used in the synthesis was ethanol (EtOH, CAS 64‐17‐5, 99.8%, Fisher Scientific). To determine a better silica to alumina ratio, a microporous silica was synthesized. The synthesis was carried out using distilled water. Initially, ethanol and water were mixed with the ANN until complete dissolution of the ANN in the mix. Following this, TEOS was added dropwise. The mix was stirred for 24 h at 20 °C, after which they were heat‐treated up to 100 °C in a controlled environment (0.5 °C/min) with a dwell time of 12 h and a 2‐h dwell time at every 20 °C. Following this, the samples were washed with water and then dried at 100 °C for further characterization. To tailor the structure of the adsorbents (aluminosilicates), the three‐stage sol‐gel parameters development was as follows. ESA Series I, a range of molar proportions of aluminum precursor, ANN, was used according to Table 1. The standard molar ratio used was 1TEOS:10 H_2_O:10EtOH.

In ESA Series II, to achieve the target pore size range, the sol‐gel formulations were adjusted by changing the solvent and alumina precursor from their original formulations. These modified formulations were as follows:

1. T‐90‐NS and T‐90‐NSNa: both formulations were based on the T‐90 mix, however, no solvent (ethanol) was used. T‐90‐NS molar ratio was 1 SiO_2_: 10 H_2_O: 0.1 Al_2_O_3_. For T‐90‐NSNa, 2 m sodium hydroxide was used to generate a basic environment to develop a more particulate structure. The molar ratio of this formulation was 1 SiO_2_: 18 H_2_O: 0.1 Al_2_O_3_: 0.462 Na(OH).
2. T‐95‐AlO(OH): the alkoxide alumina precursor, aluminum isopropoxide, was used instead of a salt‐based precursor, (ANN), following the same stepwise procedure of T‐95.

Finally, the ESA Series III investigated the dynamics of primary amines (RNH_2_) in the adsorption of CO_2_, T‐100, and T‐96.25. Formulations were grafted with (3‐aminopropyl)triethoxysilane (APTES) and designated as T‐100‐NH_2_ and T‐96.25‐NH_2_, respectively. The grafting procedure was according to the protocol adopted by Ribeiro et al.^[^ ^31^ ^]^ Briefly, the synthesized aluminosilicates were added to 105 mL of APTES solution in toluene and stirred for 6 h at 80 °C. The solvent was then filtered, followed by drying of the sample at 60 °C for 24 h.

To benchmark the performance of the carbon‐captured engineered sol‐gel materials, they were compared to a zeolite (faujasite). This zeolite was synthesized following the verified syntheses of zeolitic materials.^[^ ^37^ ^]^

### 2.2 Materials Characterization

As described in Section 2.1, ESA samples were synthesized varying three characteristics of interest: i) the alumina content, ii) the pore structure, and iii) the presence of amine functionalization. These properties were confirmed and/or quantified using the following techniques:

1. Energy Dispersive X‐ray mapping (EDX) on the aluminosilicates was conducted using an Oxford Instruments Ultim Max 170 mm^2^ silicon drift detector. This technique was used to quantify the aluminum content present in the aluminosilicate. Fourier‐transform infrared spectroscopy (FTIR) using an ATR accessory was performed using a Perkin‐Elmer Frontier spectrophotometer. The range of 600 to 4000 cm^−1^ was used with a resolution of 4 cm^−1^ and 128 scans. The FTIR was also used to record Lewis and Bronsted acidic sites using the pyridine adsorption‐desorption procedure. For this, the samples were first degassed at 100 °C for 12 h under vacuum and then exposed to pyridine vapors at 60 °C for 1 h. In the next step, the pyridine was desorbed by heating the samples for 10 min at 150 °C. The FTIR spectra of the samples that underwent the pyridine procedure were collected from 600 to 4000 cm^−1^ with a resolution of 4 cm^−1^ and 128 scans. The presence of Bronsted and Lewis acidic sites is also evidence of alumina insertion on the structure, according to Ribeiro et al.^[^ ^31^ ^]^
2. The porous structure was evaluated using nitrogen adsorption, and was conducted using Quantachrome ASiQwin. The samples were degassed at 100 °C for 12 h under vacuum. The analysis was conducted at 77K. Density functional theory (DFT) was used to evaluate the pore size distribution (pore width >1 nm), surface area, and pore volume distribution according to the respective sizes.
3. Thermogravimetric analysis (TGA) of the samples was conducted with Setsys Evolution TGA 16/18 (Setaram). The temperature ranged from 30 to 1000 °C at a rate of 10 °C min^−1^ and under nitrogen flow at 20 ml min^−1^. DTG profile was calculated as the first derivative of the thermograms collected. The concentration of amine, N, (mmol/g) was calculated using Equation (1).^[^ ^38^ ^]^
(1) $$N=\left[\frac{\%\;mass\;loss_{390-650^{\circ }C}}{100}\right]\;\left(\frac{1\;mol}{58g}\right)\left(\frac{1000\;mmol}{1\;mol}\right)$$

Where % mass loss 390–650 °C is the mass loss assessed from the DTG between the temperature range of 390–650 °C. The amine group removed from the samples at this temperature range had a molecular weight of 58 g mol^−1^ (CH_2_CH_2_CH_2_NH_2_).^[^ ^38^ ^]^

Finally, to assess the CO_2_ adsorption of samples, CO_2_ physisorption tests were conducted using Quantachrome ASiQwin. The degas procedure for the samples was the same as that of nitrogen adsorption. The analysis was conducted at 293K, and the DFT available in the software was used to calculate the pore size distribution of pore width < 1 nm (micropore). As nitrogen is not a completely satisfactory adsorptive for the assessment of micropore size distribution, CO_2_ adsorption test conducted at 273 K was carried out to determine the micropore size distribution.^[^ ^39^ ^]^ The mesopore size distribution was analyzed using nitrogen as the adsorptive gas.

### 2.3 Model Calculation Procedure

The total amount of adsorbed CO_2_, θ_*t*_, was calculated based on the UIM developed by Ng et al.^[^ ^27^ ^]^ The isotherm model was given in Equation (2):

(2) $$\theta_{t}=\sum_{i=1}^{n}\alpha_{i}\left[\frac{{\left(\frac{P}{P_{0}}.exp\left(\frac{E_{i}}{RT}\right)\right)}^{\frac{R.T}{m_{i}}}}{1+{\left(\frac{P}{P_{0}}.exp\left(\frac{E_{i}}{RT}\right)\right)}^{\frac{R.T}{m_{i}}}}\right]\;$$

where P/P_0_ was the relative pressure, P_0_ represents the saturation pressure at which maximum possible uptake by the adsorbent was studied,^[^ ^40^ ^]^ and in this research, it was atmospheric pressure. E_i_ is the peak energy value of the energy distributed in the site, R is the universal gas constant; T is the adsorption temperature (293 K for the present test); α_i_ represents the contribution of each site (Σα_i_ = 1); m_i_ is the standard deviation of each energy site, whose range also represents the surface heterogeneity.

Due to the variations in the nature of the adsorbents in terms of surface functionality and pore structure, four terms were used for the sol‐gel‐based aluminosilicates. However, only two terms were used to get the best‐fitting curve of the model for faujasite in Equation (2). Matlab R2020b was used to fit the model curve in Equation (2) and the isotherm obtained from the CO_2_ physisorption test. After the parameters (E_i_, α_i,_ and m_i_) were obtained, the energy profile (X(e)) was calculated using Equation (3). *h_fg_* is the latent heat of vaporization of CO_2_ at 20 °C. Each group of sites is collectively named from S_1_ to S_4_. For instance, group S1 is mathematically related to the first term in the summatory of Equation (2). Physically, S_1_ is the group of active sites represented by a curve with average energy E1, standard deviation m1, and percent contribution to the total surface of α1.

(3) $$X\;\left(e\right)=\sum_{i=1}^{n}\alpha_{i}\left[\frac{exp\left(\frac{e-h_{fg}-E_{i}}{m_{i}}\right)}{m_{i}.{\left[1+exp\left(\frac{e-h_{fg}-E_{i}}{m_{i}}\right)\right]}^{2}}\right]\;$$

## 3 Results and Discussion

### 3.1 Properties of Synthesized Samples

**Figure 1:**
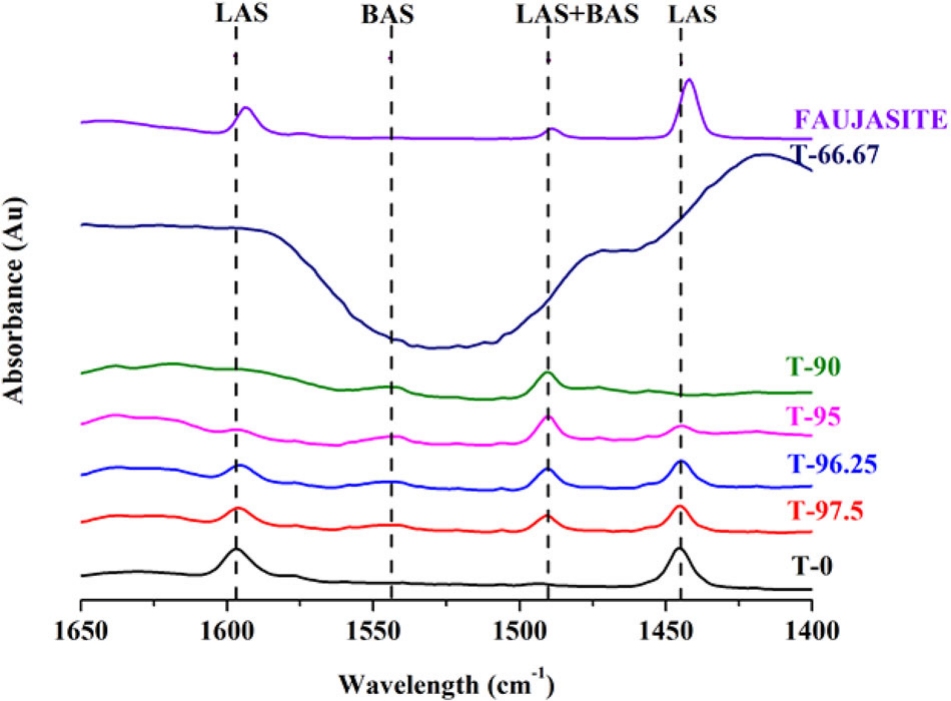
FTIR spectra of pyridine adsorption of samples an increase of alumina content.

The results of the characterization techniques are compiled in **Table** **2**. All graphs with more detailed information on these results can be found in the Supplementary Material (Figures S1–S11, Supporting Information). The Al/Si ratios found in this study were higher than the theoretical amount but followed the same trend. Moreover, the insertion of Al is also confirmed by the appearance of Lewis and Bronsted acidic sites. Both the quantity and type of acidic sites created by the Al influence the CO_2_ adsorption.^[^ ^41^ ^]^ Furthermore, most of the aluminosilicates contain both Lewis Acidic Sites (LAS) and Brønsted Acidic Sites (BAS), as shown in **Figure** **1**.

**Table 2:** Material characterization on variation with sol‐gel parameters.

ESA Series	Sample ID	Al/Si [mol%]	BET SA [m^2^ g^−1^]	t_µP_ [cm^3^ g^−1^]	t_MP_ [cm^3^ g^−1^]	Amine quantity [mmol g^−1^]	CO_2_ adsorption at 1 bar [g kg^−1^]
Series I	T‐100	0	866	0.02	0.8	0	52.9
	T‐97.5	0.07	599	0.04	0.32	0	50.6
	T‐96.25	0.08	743	0.23	0.24	0	101.0
	T‐95	0.1	647	0.05	0.26	0	56.5
	T‐90	0.20	571	0.18	0.10	0	65.0
	T‐66.67	0.92	36	0	0.06	0	93.6
Series II	T‐95‐AlO(OH)	0.1	218	0	0.49	0	59.4
	T‐90‐NS	0.15	990	0.22	0.52	0	59.7
	T‐90NSNa	0.17	291	0.07	0.15	0	32.6
Series III	T‐100‐NH_2_	0	176	0.03	0.1	0.4	48.4
	T‐96.25‐ NH_2_	0.08	218	0	0.3	0.3	13.2

Surface area values varied from 218 m^2^/g (T‐95‐AlO(OH)) to 990 m^2^/g (T‐90‐NS). Also, the proportion between micro and mesopores varied from 0% to 96% of micropores, which provides a very heterogeneous group. This variation in the micro/mesopore proportion is targeted for our analysis with the universal isotherm.

Regarding the presence of amine in the samples, it can be seen from Table 2 that incorporation of APTES into the aluminosilicate structure led to a decrease of the surface area when compared to the non‐amine grafted samples (T‐100 and T‐96.25). Furthermore, the higher the alumina content, the lower the amine grafted. This was because of fewer silanol groups to bond with amine groups.^[^ ^31^ ^]^ Theoretically, 1 mmol of amine can approximately adsorb 1 mmol of CO_2_.^[^ ^42^ ^]^ From Table 2, T‐100‐NH_2_ adsorbed three times more CO_2_ than its theoretical amount (0.4 mmol of CO_2_/g). This confirms that CO_2_ adsorption of amine‐grafted material is also dependent on the pore structure of the material.

### 3.2 Isotherm Modeling

**Figure 2:**
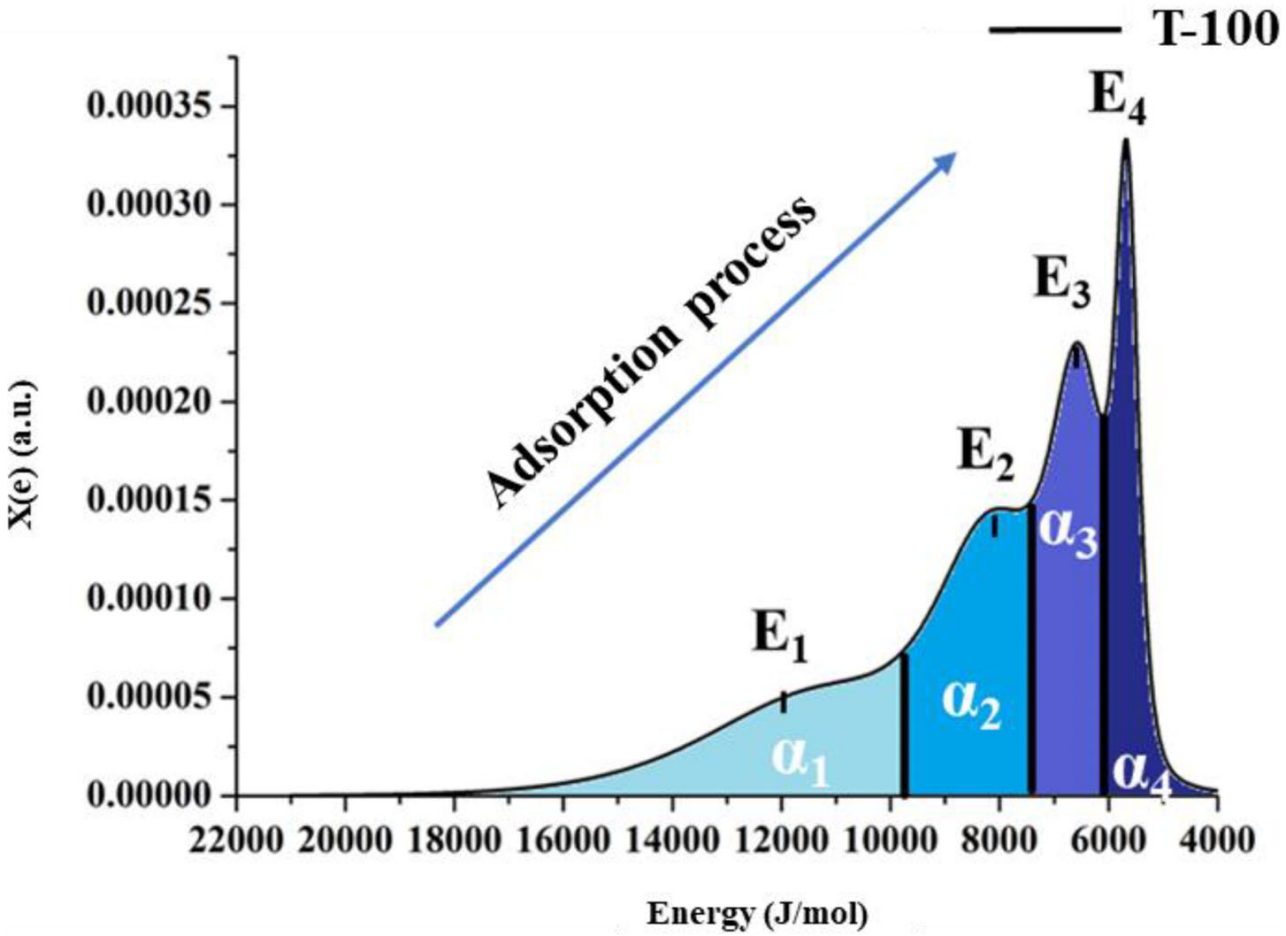
T‐100′s energy profile in relation to CO_2_ adsorption. The graph shows four adsorption patches from high to low energy and the direction of the adsorption. The area under the curve αi (α_1_ to α_4_) represents the surface coverage of the respective sites.

The CO_2_ adsorption isotherms obtained were modeled using Equations (2) and (3). It was identified that the synthesized sol‐gel aluminosilicates required four terms in Equation (2) to get the best fit curve for the experimental CO_2_ adsorption data. This is an innovative aspect of the present work, since the original use of the UIM model proposed the use of only two terms for the regression of the experimental data.^[^ ^27^ ^]^ According to the identified number of sites for the samples, the surface of the samples was divided into respective adsorption patches. **Figure** **2** shows the energy distribution curves of sample T‐100. The surface of T‐100 is divided into four adsorption patches, S_1_ to S_4_. Each adsorption patch has a threshold energy, E_i_, with an overall surface coverage of α_i_. The standard deviation or the range of energies, m_i_, represents the heterogeneity of each group of adsorption sites. The sum contribution of the four adsorption sites is represented as the total area under the curve (α_1_ to α_4_).

The adsorption mechanism initiates by filling the sites of high energy (S_1_ and S_2_) at low pressure, followed by adsorption at lower energy sites (S_3_ and S_4_) as the partial pressure increases.^[^ ^27^, ^31^, ^43^ ^]^ The saturation pressure for CO_2_ was 64.5 bar, however, only up to 1 bar is considered in this study, as the intention was to investigate the high‐energy adsorption sites, which are at lower pressure. This lower pressure was chosen because this is the condition more approximate to the application of engineered aluminosilicates to CO_2_ capture in cement materials.

#### 3.2.1 Influence of Alumina Content

**Figure 3:**
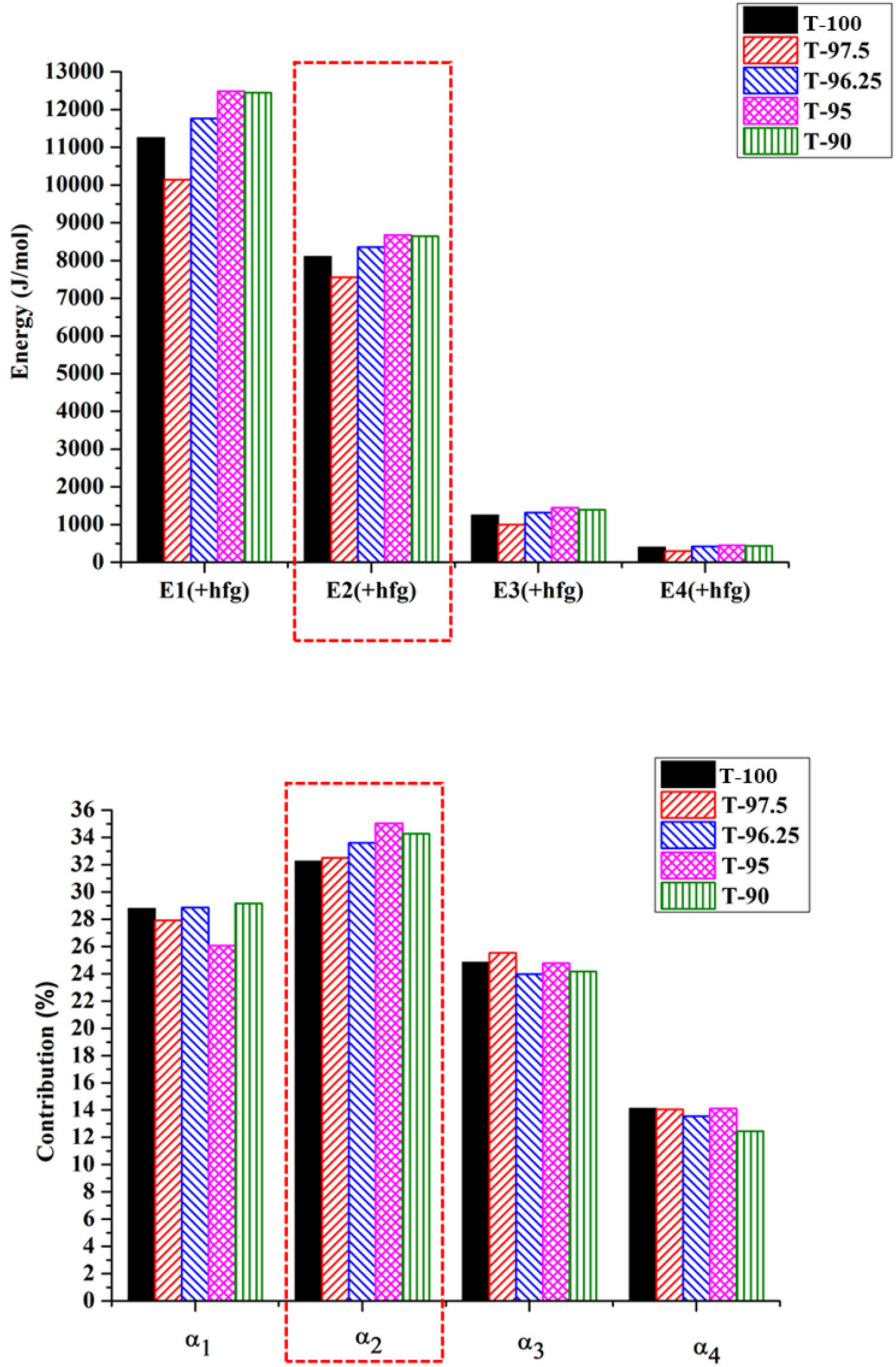
Parameters of the universal isotherm modeling for samples comparing the influence of alumina within the structure of adsorbents. The highlighted parameters (E_2_ and *α_i_*_2_) are influenced by the alumina content. *h_fg_* is the latent heat of vaporization of CO_2_ at 20 °C.

**Figure** **3** shows the E_i_ and α_i_ values of samples (T‐100, T‐97.5, T‐96.25, T‐95, and T‐90) selected for evaluating the influence of alumina content on CO_2_ adsorption. In general, E_1_, E_2_, α_1,_ and α_2_ were higher for samples with alumina content when compared to T‐100. Sample T‐97.5 showed a lower E_2_ value compared to T‐100; however, this energy value can still be considered within the same range, as the standard deviation m_2_ ranges from 500–700 J/mol (values available in Table S1, Supporting Information). Furthermore, T‐97.5 presents higher surface coverage of CO_2_ over adsorption site 2, α_2,_ when compared to T‐100. The increase of E_2_ and α_2_ can be theoretically supported with the LAS+BAS sites at 1491 cm^−1^ in Figure 2. According to the versatile amorphous silica‐alumina (ASA) surface model by Leydier et al.^[^ ^44^ ^]^ the pseudo‐bridging silanol (PBS) groups created by alumina sites (PBS‐Al) are responsible for the highest adsorption of C═O. The PBS‐Al sites are the LAS+BAS sites in the aluminosilicate materials (at 1491 cm^−1^ in the FTIR spectrum, Figure 2), which are created by Al^IV^ or Al^V^. With an increase in the Al/Si ratio, the Al^IV^ species tends to condense to form Al^V^ or alumina.^[^ ^45^ ^]^ This will indeed reduce the CO_2_ adsorption. Thus, the S_1_ and S_2_ adsorption sites are associated with the alumina content.

#### 3.2.2 Aluminosilicate Pore Structure

**Figure 4:**
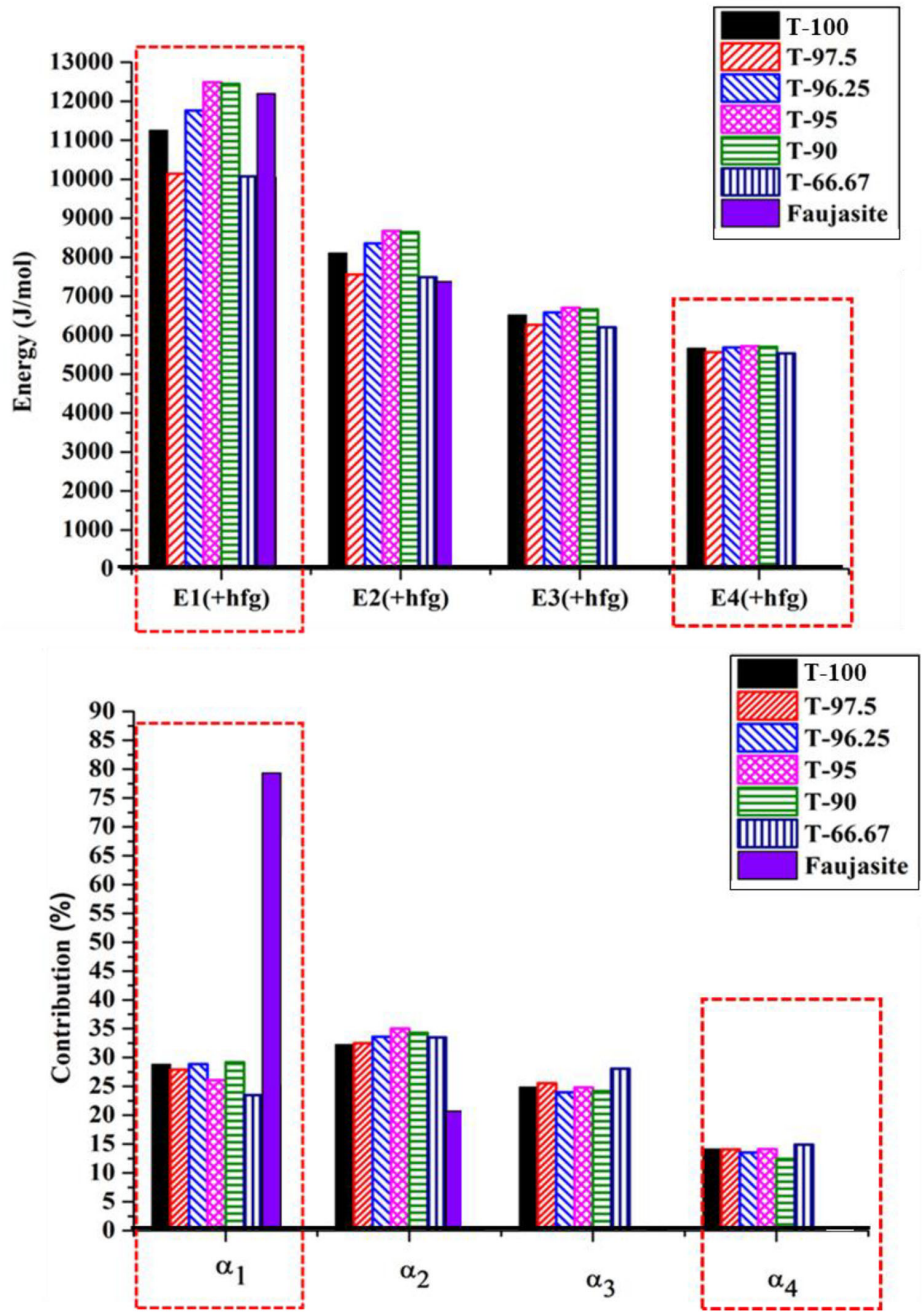
Parameters of universal isotherm modeling for samples comparing the mesopores and micropores in the adsorbent structure an increase in alumina content. *h_fg_* is the latent heat of vaporization of CO_2_ at 20 °C.

**Figure 5:**
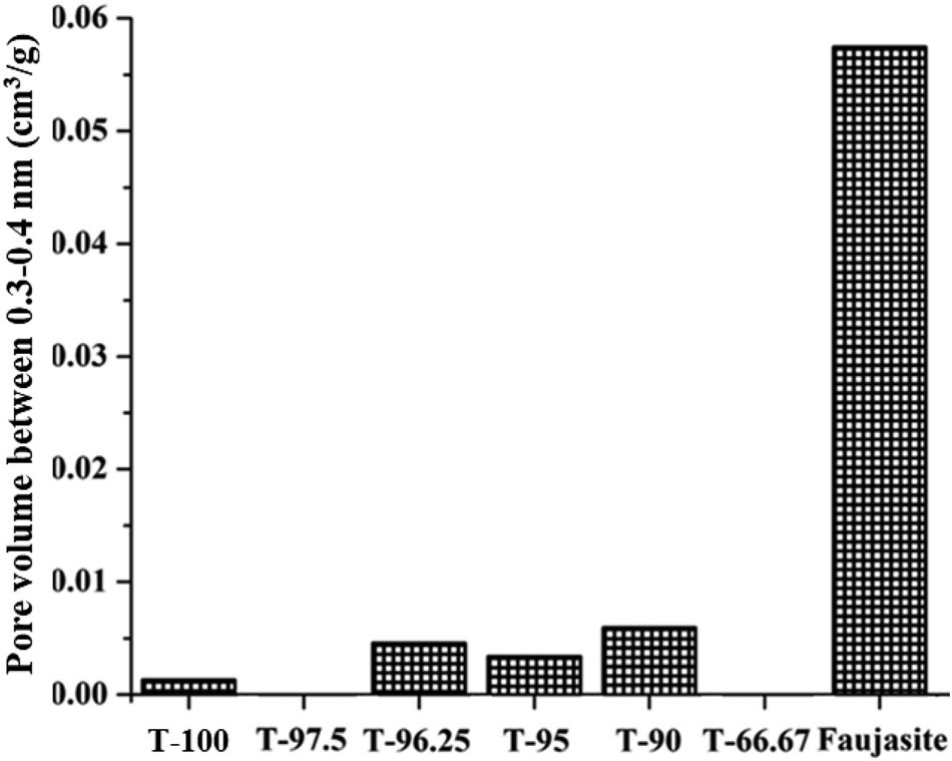
Ultramicropores volume of the pore size in the range of 0.3–0.4 nm for an increase in alumina content. Values for T‐97.5 and T‐66.67 were negligible.

The first variable examined was the increase in alumina content within the adsorbent. **Figure** **4** displays the E_i_ and α_i_ of the sample group selected to evaluate the influence of pore structure on alumina content increase in the adsorbent. **Figure** **5** illustrates the ultramicropore volume of the chosen samples in the study. It was observed that as the ultramicropores of the aluminosilicates increased, there was also an increase in E_1_ and α_1_. Therefore, the universal isotherm indicated that to enhance CO_2_ adsorption in aluminosilicates, the ultramicropores need to be increased.

**Figure 6:**
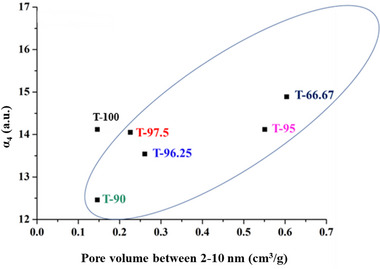
Comparison between mesopore volume in the pore size range of 2–10 nm and the α_4_ parameter of the universal isotherm model for the samples in the study on the increase in alumina.

**Figure** **6** presents the relationship between mesopore volume in the 2–10 nm range and α_4._ It was found that the mesopore volume of the aluminosilicates remained comparable across samples. The values of E_4_ and α_4_ were also similar, suggesting mesopores influence the lowest energy sites S_4_. Additionally, T‐66.67 showed a marginal increase in mesopore volume, which corresponded to a rise in α_4._

**Figure 7:**
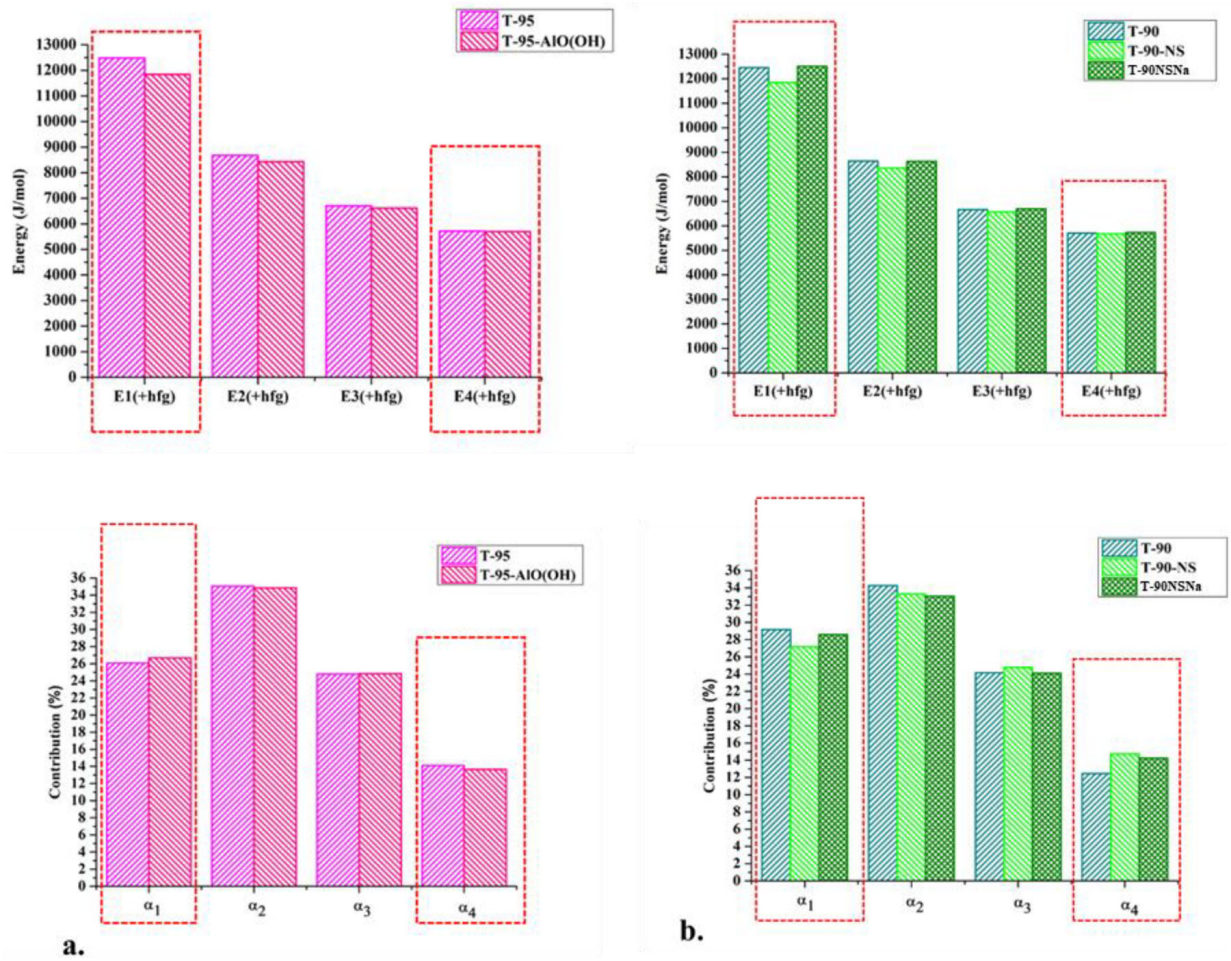
Parameters of universal isotherm modeling for samples comparing the mesopores and micropores in the adsorbent structure on; a) variation in the alumina precursor; b) variation on solvent addition.

**Figure 8:**
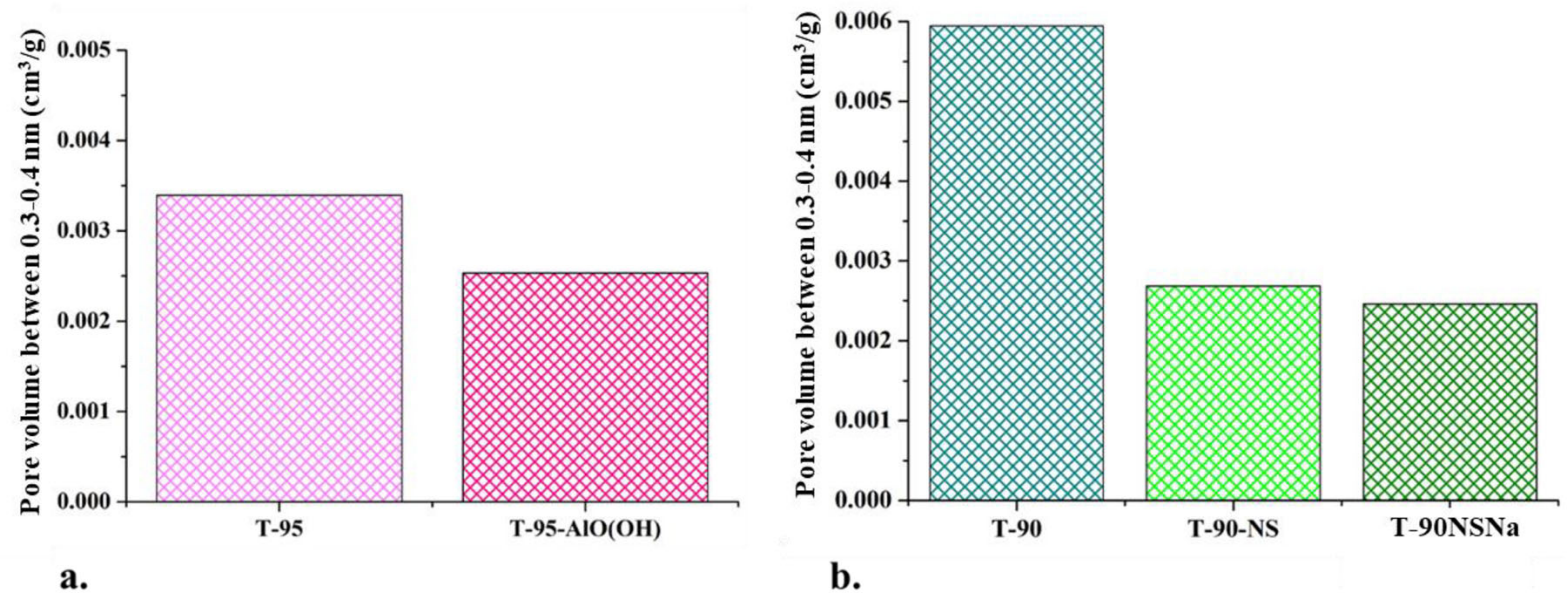
Ultramicropores volume of the pore size in the range of 0.3–0.4 nm for the samples in the study; a) variation with alumina precursor, b) variation with solvent.

The next sol‐gel variable examined was the choice of alumina precursor and the introduction of a solvent, both intended to explore how these factors impact pore structure and thus CO_2_ adsorption performance. **Figure** **7** presents the E_i_ and α_i_ values for a selected group of samples, highlighting the effect of pore structure on CO_2_ capture, while **Figure** **8** displays the volume of ultramicropores for these samples.

A clear, strong correlation was found between the E1 value and the ultramicropore volume. For example, when the solvent was varied, the T‐95 sample exhibited a greater ultramicropore volume compared to T‐95‐AlO(OH), which corresponded to its higher E1 value. Further comparison within the group of samples altered by solvent addition showed that T‐90 experienced a slight increase in ultramicropore volume, which was matched by a corresponding rise in E1.

**Figure 9:**
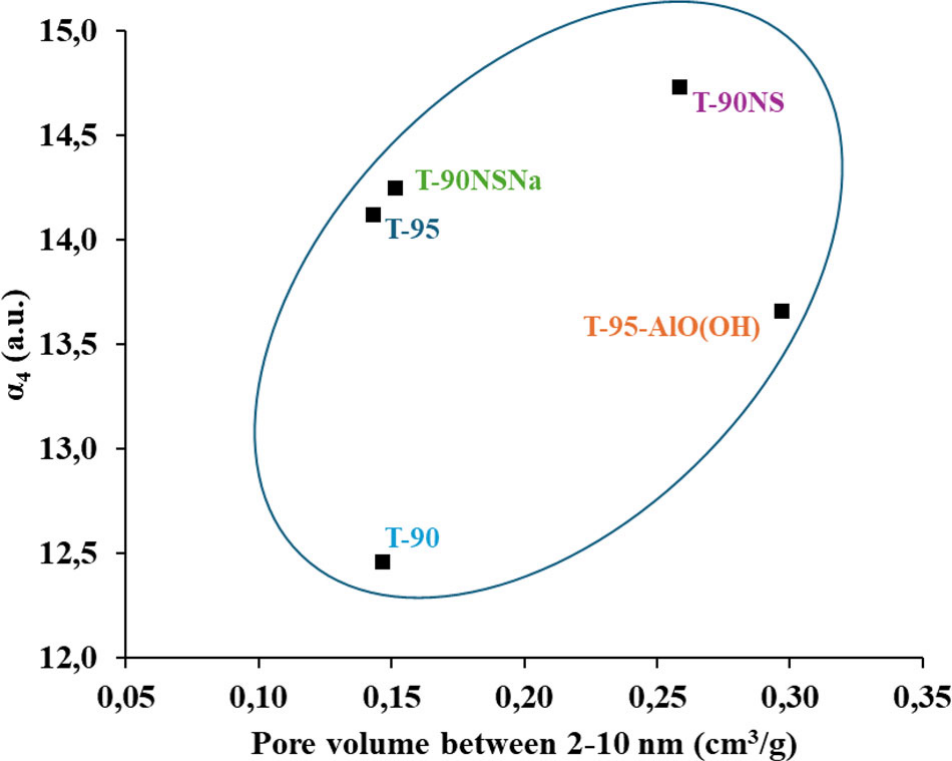
Comparison of mesopore volume within the pore size range of 2–10 nm and the α4 parameter of the universal isotherm model for the samples in the study, comprising the variation with alumina precursor and solvent.

**Figure** **9** shows the relation between the mesopore volume in the range 2–10 nm and α_4_ of the selected set of samples. For both sets (variation with alumina precursor and addition of solvent), the samples with higher mesopore volume showed higher α_4_ values. This suggests that the influence of mesopores occurs in S_4_ sites.

The results were consistent with Polanyi's adsorption theory,^[^ ^46^ ^]^ which states that the surface of the adsorbent creates a potential field of attraction. The potential field intensifies as the adsorbate gets closer. Pores smaller than 1.0 nm were highly effective in CO_2_ adsorption at atmospheric pressure. This is based on the kinetic diameter of the CO_2_ molecule, which is 0.33 nm.^[^ ^47^ ^]^ Furthermore, high CO_2_ capacity was mainly attributed to the presence of small micropores (<0.8 nm) or ultramicropores (<0.7 nm). The mesopores promote the diffusion and transport of CO_2_ to the micropores.^[^ ^48^ ^]^ The results were consistent with the theory of CO_2_ adsorption capacity associated with the theory of interconnected micro‐mesoporosity for enhanced CO_2_ adsorption of the materials at atmospheric pressure.^[^ ^47^, ^49^, ^50^ ^]^ Furthermore, it was identified by Durá et al.^[^ ^49^ ^]^ that CO_2_ is physisorbed into the micropores, most of which are only accessible through the mesopores. Hence, confirming recent studies from Jin et al.^[^ ^51^ ^]^ for strong interactions, ultramicropores were needed; however, the mesopores helped in the diffusion of the CO_2_ to the micropores. This was the reason T‐96.25 showed an improved CO_2_ adsorption when compared to the other synthesized aluminosilicates (Figures 5 and 6).

#### 3.2.3 Role of Amine Grafting

**Figure 10:**
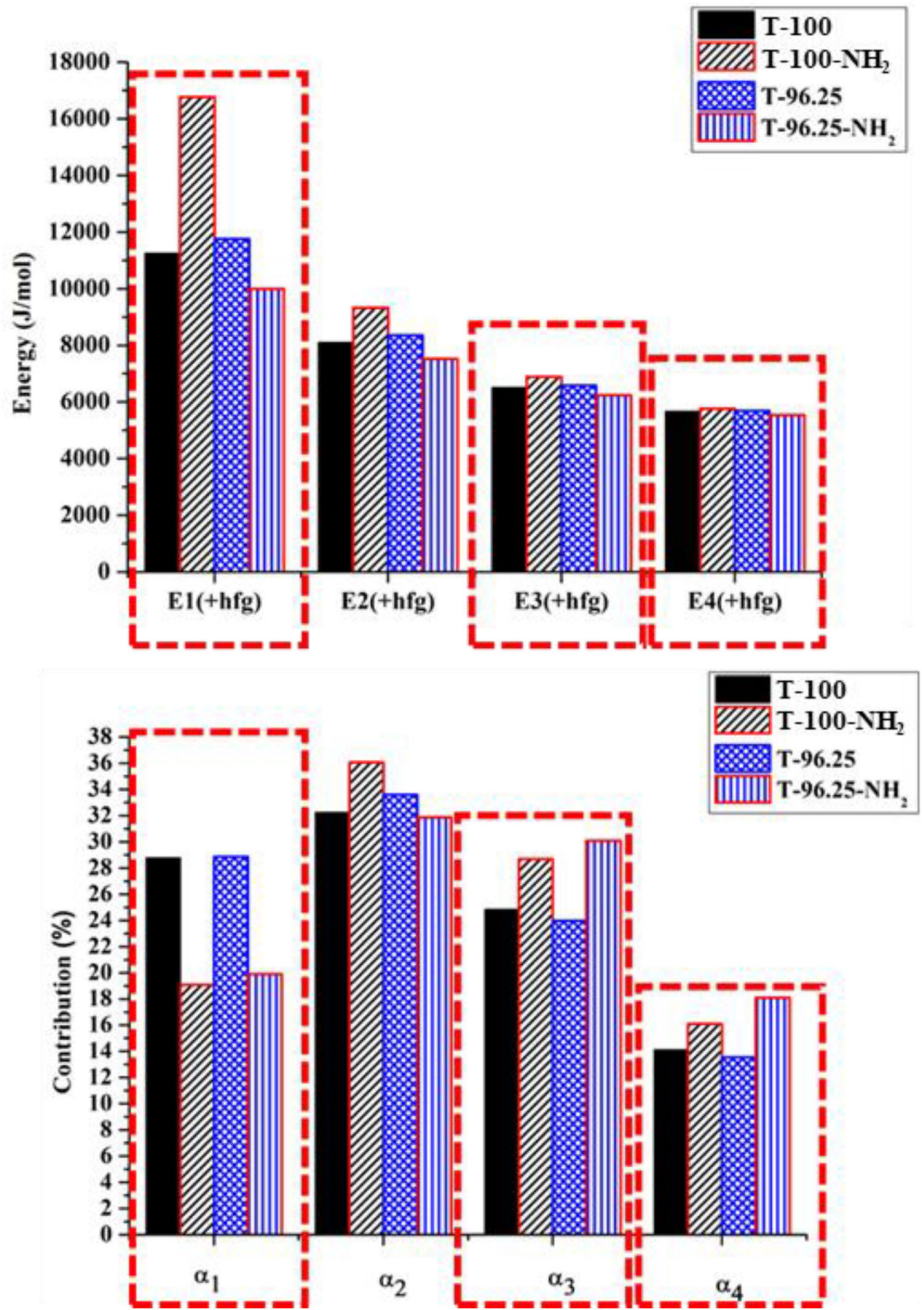
Parameters of universal isotherm modeling comparing the samples that were amine grafted.

**Figure 11:**
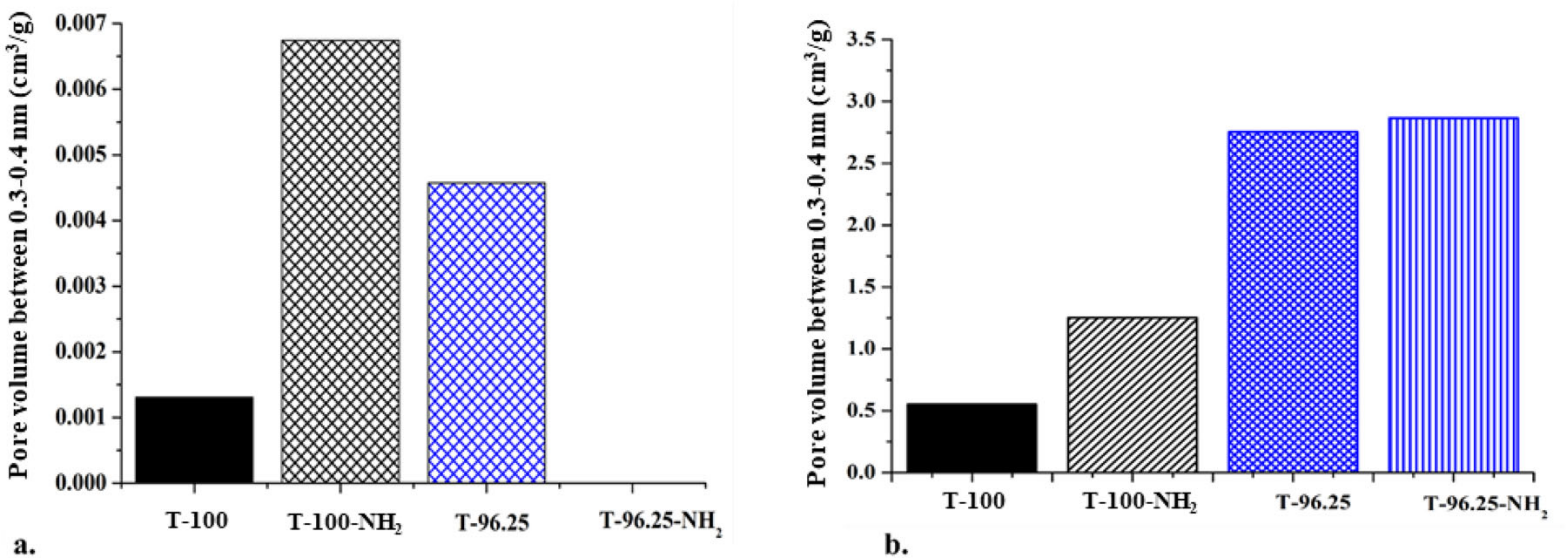
Comparison of pore volume in the grafted samples. a) Ultramicropores volume between 0.3 and 0.4 nm; b) mesopore volume within the pore size range of 2–10 nm for amine‐grafted samples.

**Figure** **10** shows E_i_ and α_i_ of the group of samples selected for the evaluation of influence of amine grafted on the aluminosilicates. α_3_ was higher for the amine‐grafted samples when compared to the non‐grafted samples, suggesting the influence of amine on S_3_ sites. The pore volume in the ultramicropores range and mesopore range of the selected samples is shown in **Figure** **11**.

At low pressure, T‐100‐NH_2_ exhibited the highest CO_2_ adsorption compared to the other samples (Table 2). This behavior indicates the presence of high‐energy sites. Supporting this observation, Figure 11a shows that the maximum volume of ultramicropores, specifically in the range of 0.3–0.4 nm, follows the order: T‐100‐NH_2_ > T‐96.25 > T‐100. Therefore, the E_1_ sites correspond to the ultramicropores within this size range. The contribution α1 of T‐100‐NH_2_ to S1 sites is notably low, primarily due to the blockage of some micropores by APTES, which resulted in the formation of larger mesopores during the grafting procedure (see Figure S8, Supporting Information). Interestingly, the α_1_ values for the grafted samples are lower in comparison to the non‐grafted samples. This suggests that while the ultramicropores influence the highest energy sites due to the diminished contribution of S_1_ sites, the overall CO_2_ uptake remains low at atmospheric pressure. Conversely, T‐96.25‐NH_2_ exhibited the highest mesopore volume within the 2–10 nm range (refer to Figure 11b), reinforcing the idea that the mesopores were the controlling factor for S_4_, which exhibited the lowest adsorption energy.

### 3.3 Mechanism of CO_2_ Adsorption at Atmospheric Conditions

The findings of this study align with the CO_2_ adsorption mechanisms outlined by Romero et al.^[^ ^52^ ^]^ The adsorption process comprises two stages: i) *diffusive mechanism*: this initial stage involves the transport of CO_2_ to the active sites within the adsorbent's pores. At low pressure, CO_2_ diffuses into the pore structure through the mesopores, and subsequently plugs onto the ultramicropores at the S1 sites of the aluminosilicates; ii) *adsorption mechanism*: once the ultramicropores are filled, CO_2_ molecules are attracted to nearby alumina at the S_2_ sites. As the partial pressure increases, some CO_2_ is also drawn to the mesopores at the S_4_ sites until equilibrium is achieved. In cases involving amine grafting, the NH_2_ groups at the S_3_ sites attract CO_2_ during this second stage as the pressure rises.

**Figure 12:**
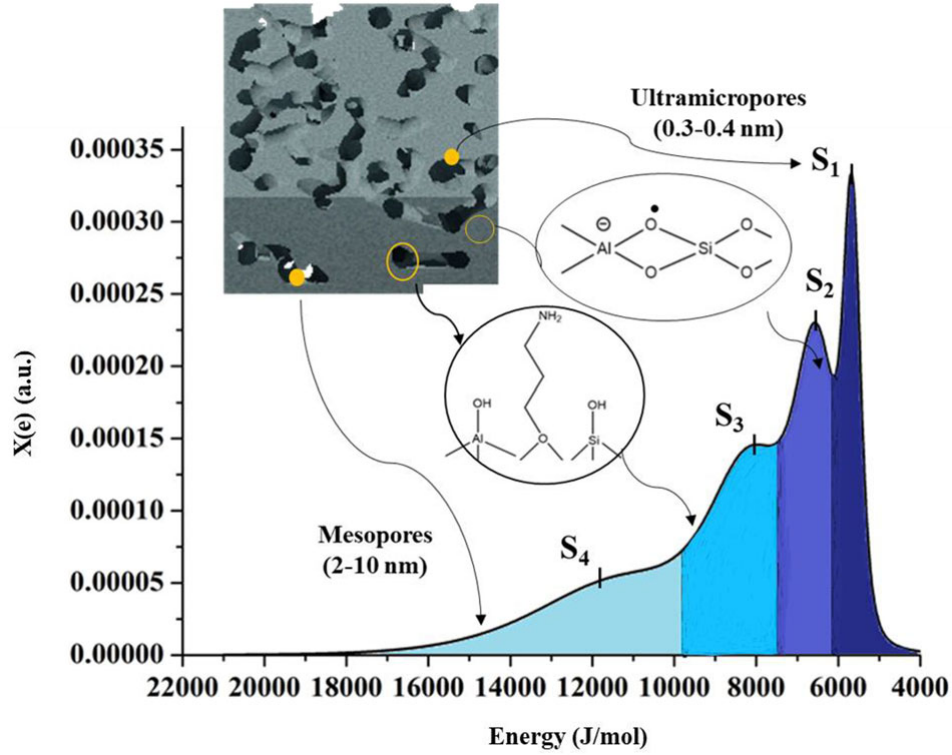
Schematic representation of the dynamics of adsorption sites and of the selected variables.

**Figure** **12** illustrates the energy profile obtained from the UIM regarding CO_2_ adsorption on aluminosilicates. It demonstrates that ultramicropores are the most influential sites at low pressures, which is particularly relevant for the application of these materials in cement pastes under atmospheric CO_2_ conditions.

### 3.4 Evaluation of Microporous Benchmark Material

Since the results presented in the previous sections highlighted the critical role of micropores, particularly ultramicropores, in providing the highest‐energy sites for CO_2_ adsorption, a benchmark material with a well‐established microporous structure, Faujasite, was selected for further analysis.^[^ ^53^, ^54^ ^]^ It is important to note that these high‐energy adsorption sites are particularly relevant to the present discussion, as they exhibit enhanced activity at low pressures and low CO_2_ concentrations, such as those encountered in atmospheric air for the curing of cement. The characterization data obtained for this material are summarized in **Table** **3**.

**Table 3:** Summary of results for the characterization and UIM modeling of Faujasite.

Characterization		Modeling	
Al/Si [mol%]	0.54	E_1_ (+h_fg_)	12196
BET SA [m^2^ g^−1^]	1913	E_2_(+hfg)	7372
t_µP_ [cm^3^ g^−1^]	0.15	α_1_ (%)	79.32
t_MP_ [cm^3^ g^−1^]	0.37	α_2_ (%)	20.68
Amine quantity [mmol g^−1^]	0	m_1_	1658
CO_2_ adsorption at 1 bar [g kg^−1^]	172.7	m_2_	760

In Table 3, the high specific surface area of the material, determined via nitrogen adsorption, is noteworthy (1913 m^2^/g against 3 –990 m^2^/g for ESA samples), as is its considerable CO_2_ adsorption capacity (173 g kg^−1^ against 13–101 g kg^−1^ for ESA). The presence of complementary mesopores in the structure can also be observed from the data and is further confirmed by the pore size distribution (Figure S7, Supporting Information). However, despite mesopores accounting for 71% of the total pore volume, their presence did not alter the overall shape of the N_2_ and CO_2_ adsorption isotherms, which retained the type I profile of the IUPAC classification^[^ ^55^ ^]^ consistent with the predominance of microporosity (Figure S4, Supporting Information).

To assess the performance of the UIM modeling for this material, we applied it to the CO_2_ adsorption isotherm of Faujasite. Notably, in contrast to the ESA samples, which required four terms to achieve an R^2^ value greater than 0.999, the best fit for Faujasite was obtained using only two terms, yielding an R^2^ value of 0.996. Consequently, only the parameters for sites S_1_ and S_2_ are reported in Table 3. This reduction in the number of terms indicates a lower heterogeneity of adsorption sites in Faujasite compared to the ESA samples, consistent with its well‐defined crystalline microporous structure.

The values for E_1_ and E_2_ given in Table 3 are very similar to those found for the ESA samples shown in Figure 4 (E_1_ = 12.2 kJ mol^−1^ against 10–16 kJ mol^−1^ for ESA; E_2_ = 7.4 kJ mol^−1^ against 7–9 kJ mol^−1^ for ESA samples), indicating that the energy levels of the adsorption sites are comparable across all materials. This similarity allows for a direct comparison between ESA and Faujasite, even though the fitting required a different number of terms for each. Despite the similar energy levels, the contribution of the highest‐energy sites, α_1_, is ≈80% in Faujasite, compared to only 20–30% in the ESA samples (as shown in Figure 4). This indicates that high‐energy sites occupy a much larger fraction of the surface area in Faujasite than in ESA, which is consistent with the higher micropore content of this material, a key hypothesis of the present study. Thus, these results confirm the influence of micropore presence on the highest‐energy adsorption sites (S_1_). It is important to emphasize that the findings presented here are based on thermodynamic data; kinetic effects and diffusion processes, which also play a significant role in adsorption, as discussed in Section 3.5, are not addressed in by these results.

Based on the findings obtained from the application of the UIM model to these systematically selected samples, it can be concluded that the ideal adsorbent for application in carbon capture in concrete would be one that maximizes the presence of active sites, such as Lewis and Brønsted sites provided by alumina, while also maximizing the presence of ultramicropores. This understanding is significant as it provides the knowledge base and methodological tools required to engineer new materials for this application, thereby informing and guiding the next steps in research within this field.

## 4 Conclusion

This study established a foundational understanding of CO_2_ adsorption dynamics in aluminosilicate materials by employing the Universal Isotherm Method (UIM). The influence of pore architecture on adsorption performance was benchmarked using faujasite, revealing critical contributions from three parameters: alumina integration within the silica framework, pore geometry, and amine functionalization.

UIM analysis demonstrated the pronounced effect arising from the interplay of ultramicropores (0.3–0.4 nm), alumina incorporation, and amine grafting. The ultramicropores provide high‐energy adsorption sites (S_1_), which are essential for the efficient capture of CO_2_ at low concentrations. In contrast, alumina incorporation and amine functionalization modulate lower‐energy sites (S_2_, S_3_), which become operative at higher pressures, thereby enhancing the overall CO_2_ uptake capacity.

Unlike conventional modeling approaches, UIM provided a nuanced energy landscape of surface interactions, enabling a more predictive understanding of how structural modifications influence adsorption efficiency. Notably, enhancing the contribution of S_1_ sites, exemplified in faujasite, emerged as a key strategy for maximizing CO_2_ uptake under ambient conditions.

These findings offer a transferable framework for the rational design of high‐performance CO_2_ adsorbents, with potential applications spanning low‐carbon construction and advanced membrane technologies. The insights gained here pave the way for accelerated innovation in carbon capture systems engineered for real‐world environmental impact.

## Conflict of Interest

The authors declare no conflict of interest.

## Supporting information



Supporting Information

## Data Availability

The data that support the findings of this study are available from the corresponding author upon reasonable request.
