# Supplementary material for: From Pores to Pavement: Advanced Modeling of Aluminosilicates for Scalable Carbon Capture in Concrete
Source: Adv Sci (Weinh). 2025 Nov 7:e17317. Online ahead of print. doi: 10.1002/advs.202517317 (PMC13325500; doi:10.1002/advs.202517317)
Supplement: Supplementary file 1 — Supporting Information [file ADVS-9999-e17317-s001.docx]

**SUPPLEMENTARY MATERIAL**

FROM PORES TO PAVEMENT: ADVANCED MODELLING OF ALUMINOSILICATES FOR SCALABLE CARBON CAPTURE IN CONCRETE


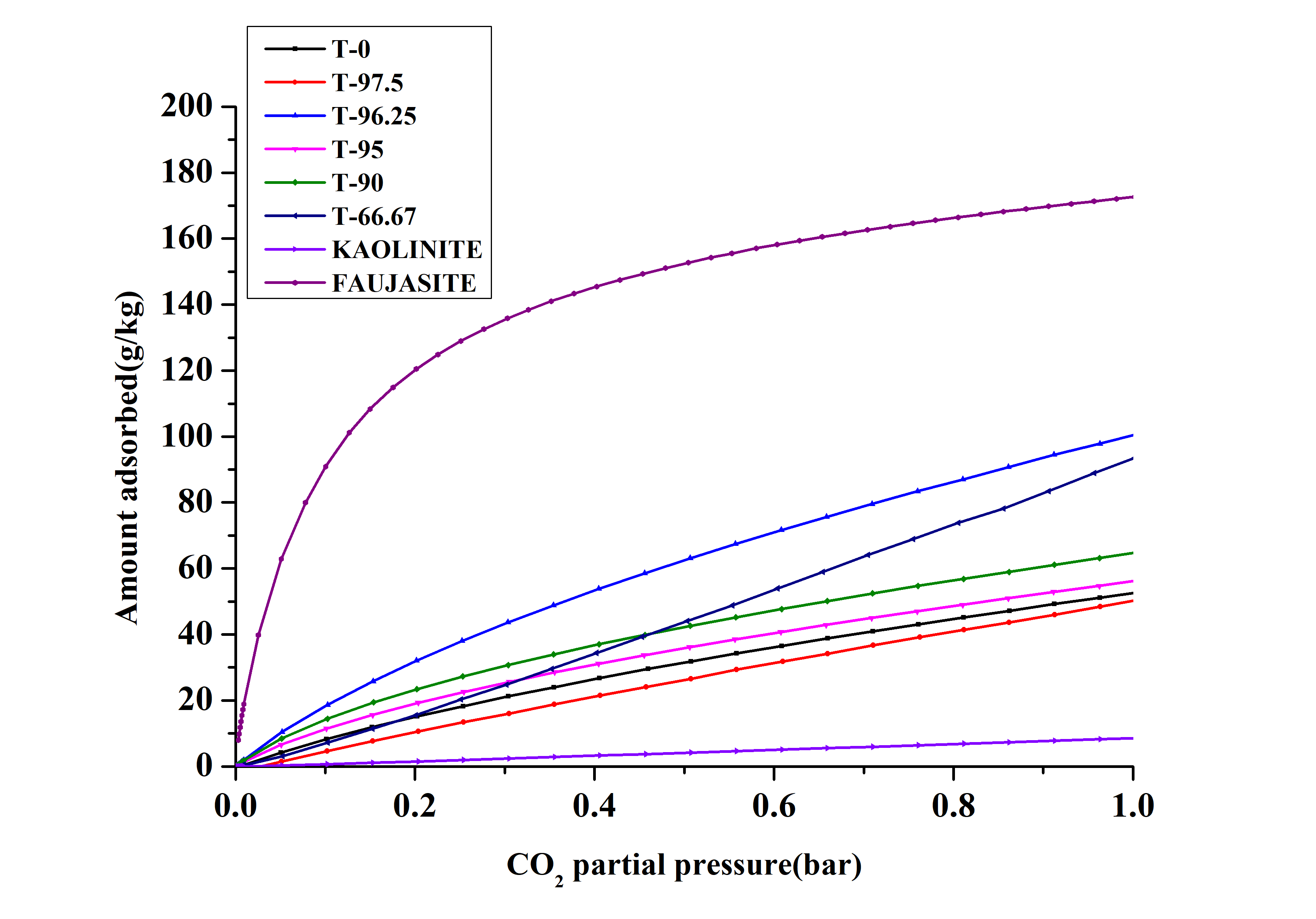


**Figure S1:** CO_2_ adsorption of samples on increase in alumina content


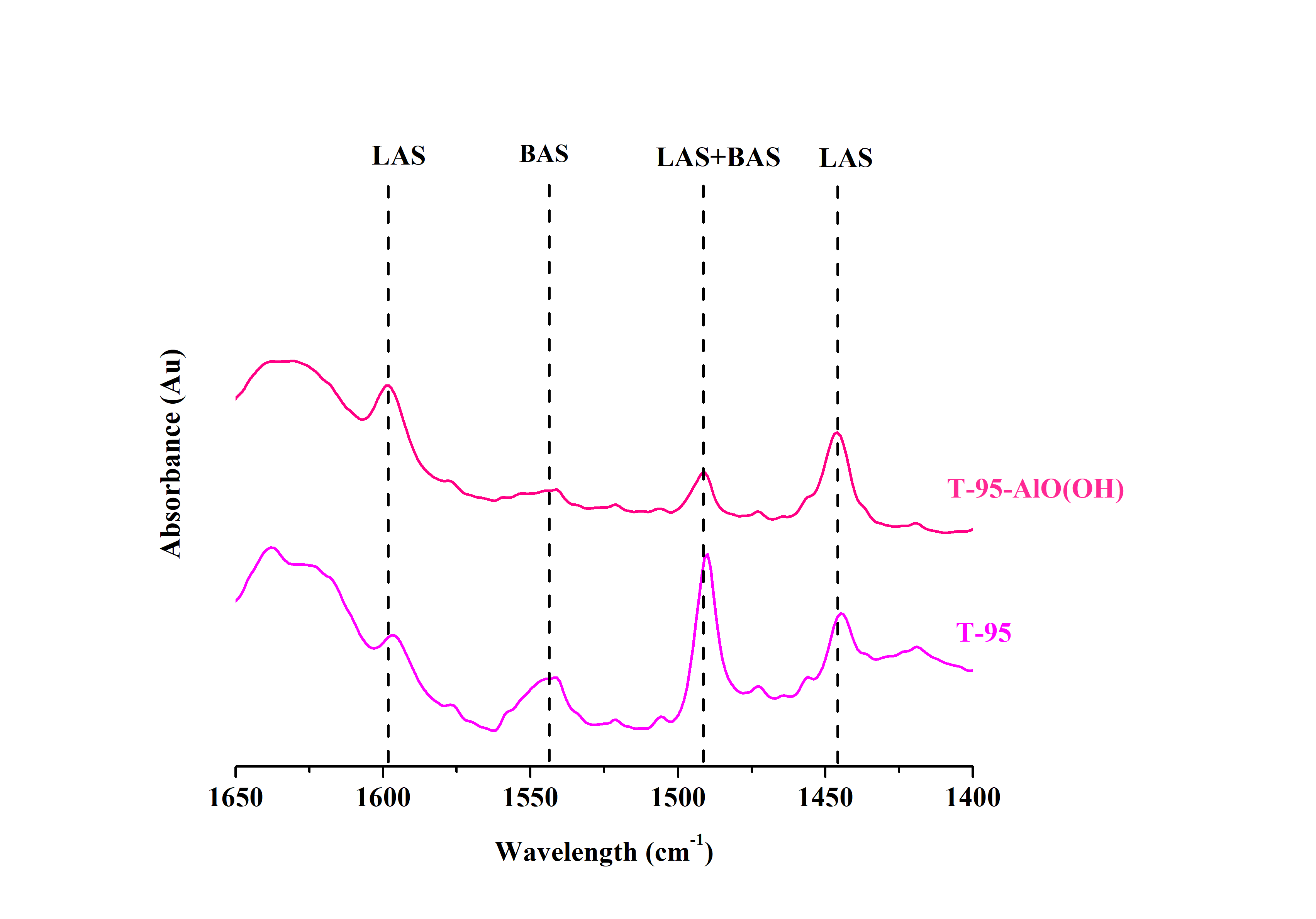


**Figure S2:** Pyridine adsorption on the variation of alumina precursor


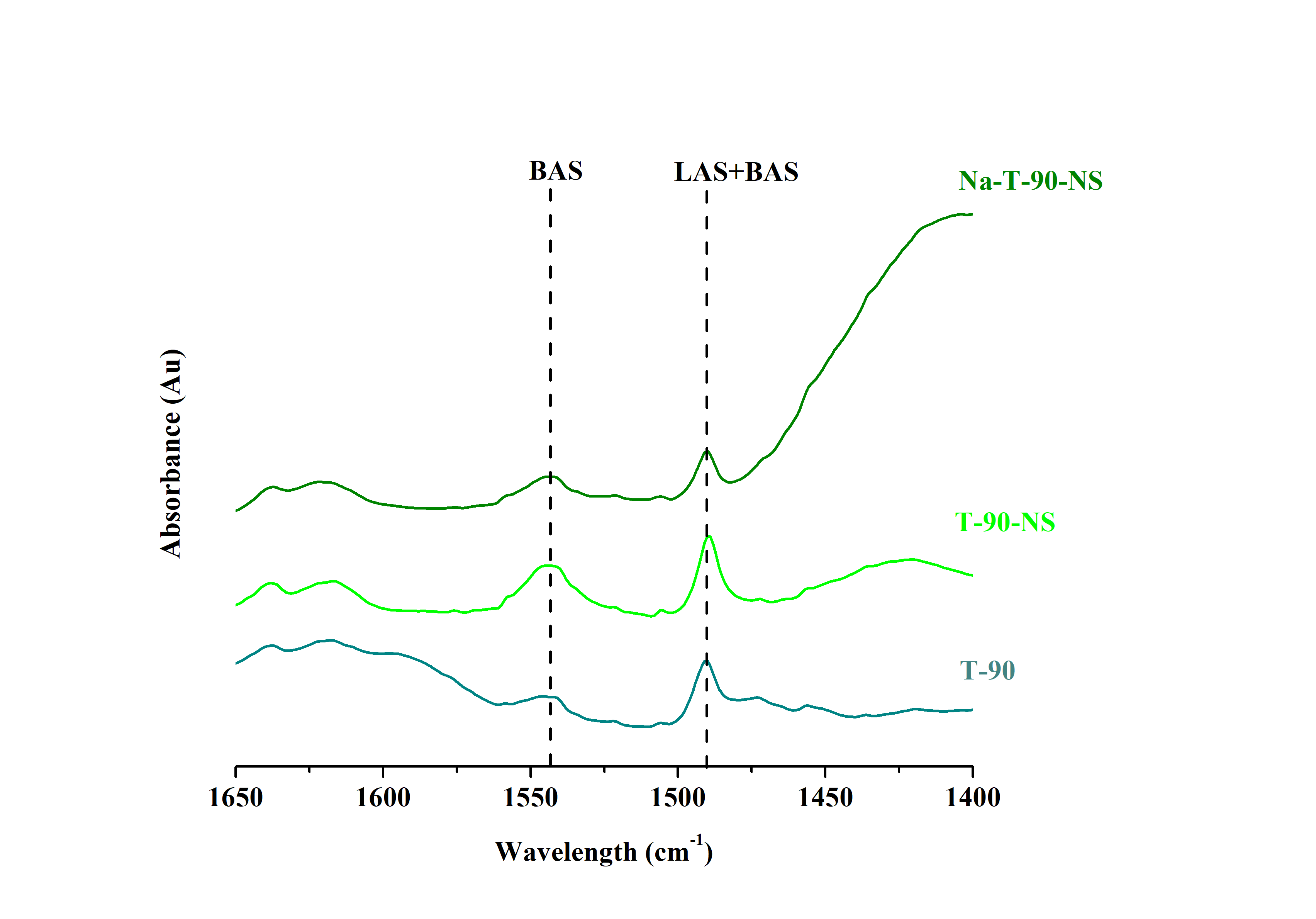


**Figure S3**: Pyridine adsorption on the addition of solvent


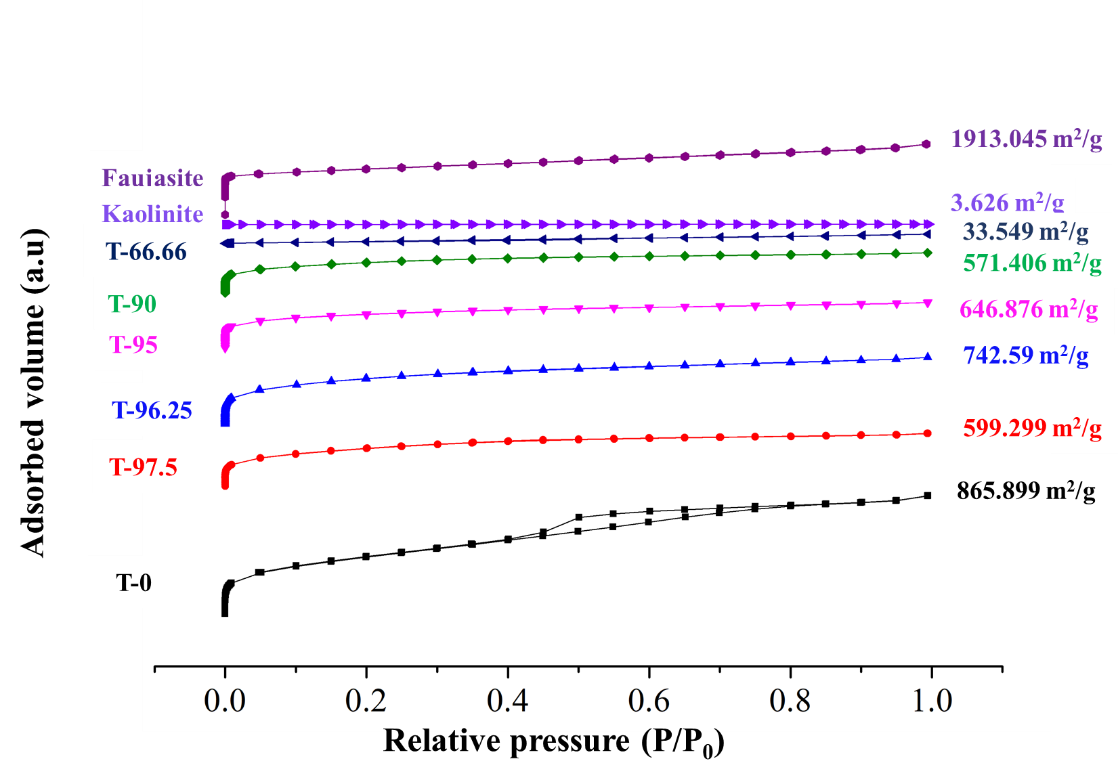


**Figure S4**: Isotherms from nitrogen adsorption on increasing alumina content


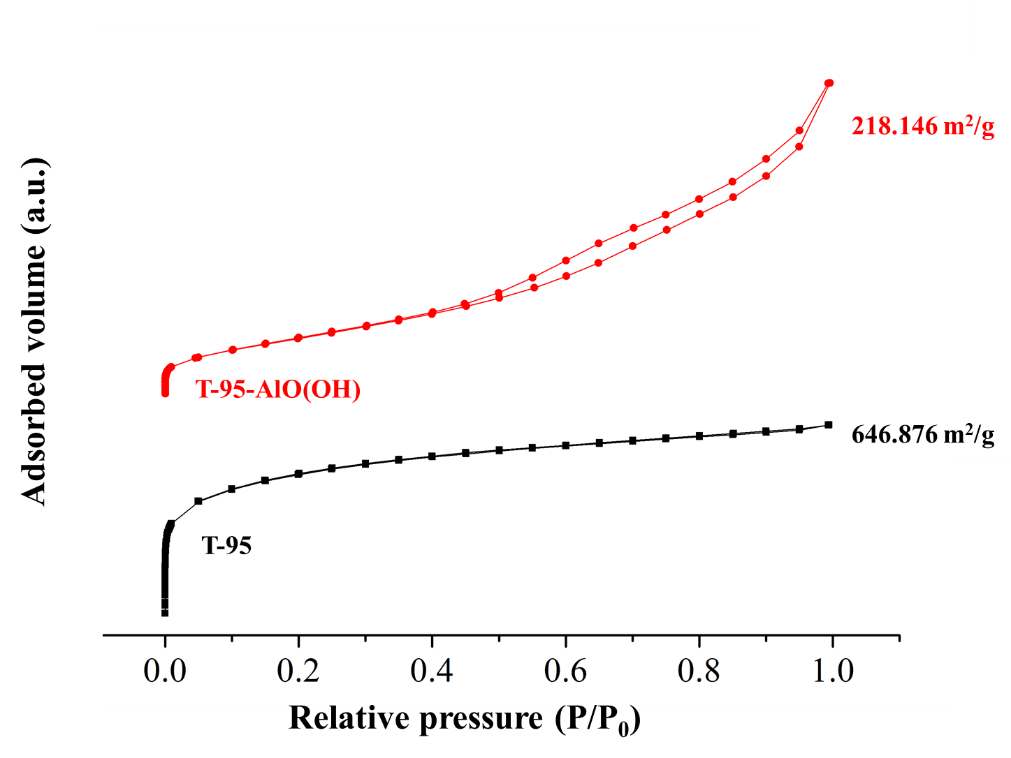


**Figure S5**: Isotherms for nitrogen adsorption varying alumina precursor


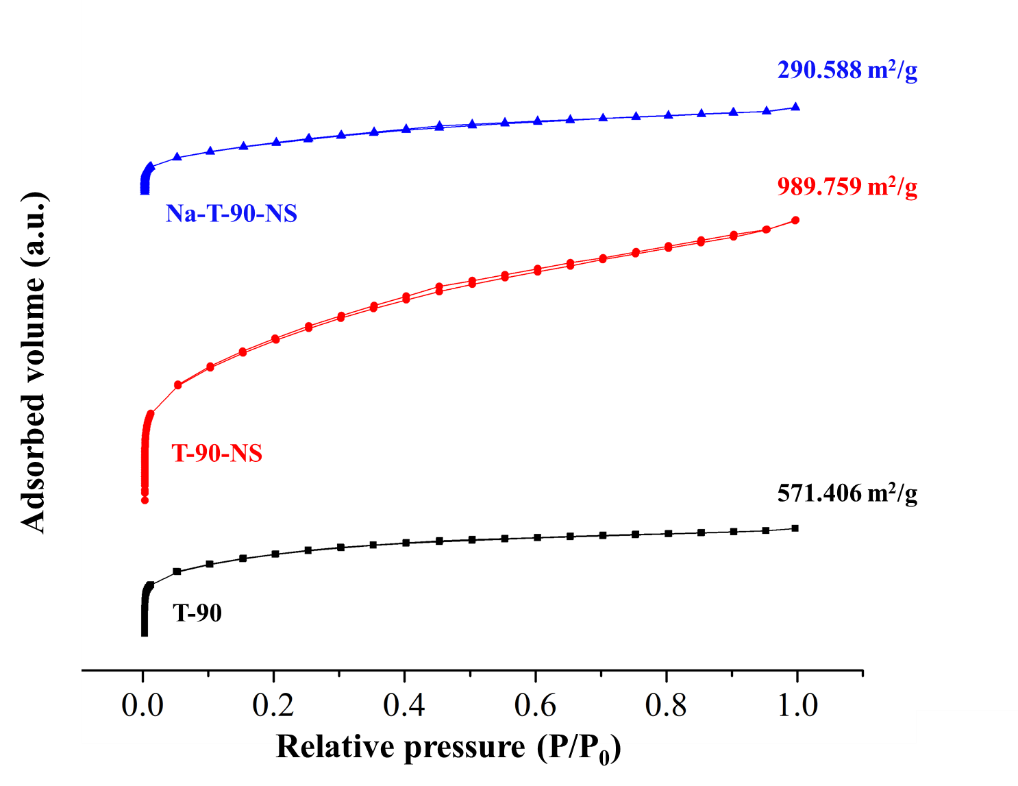


**Figure S6**: Isotherm for nitrogen adsorption on varying solvent


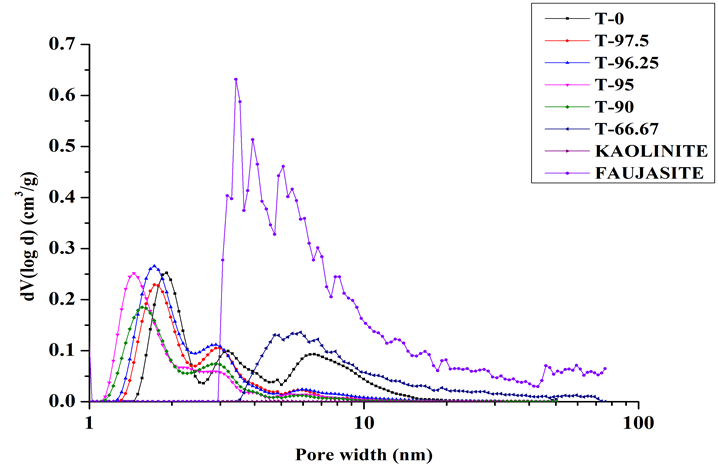


**Figure S7**: Pore size distribution of aluminosilicates on increasing the alumina content


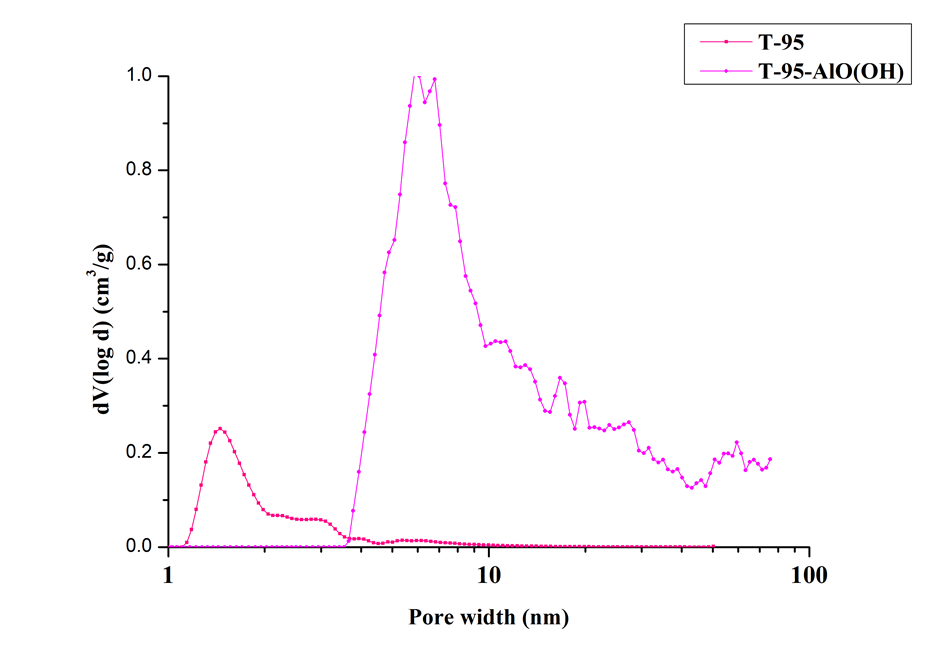


**Figure S8**: Pore size distribution of samples on variation of alumina precursor from salt-based to alkoxide


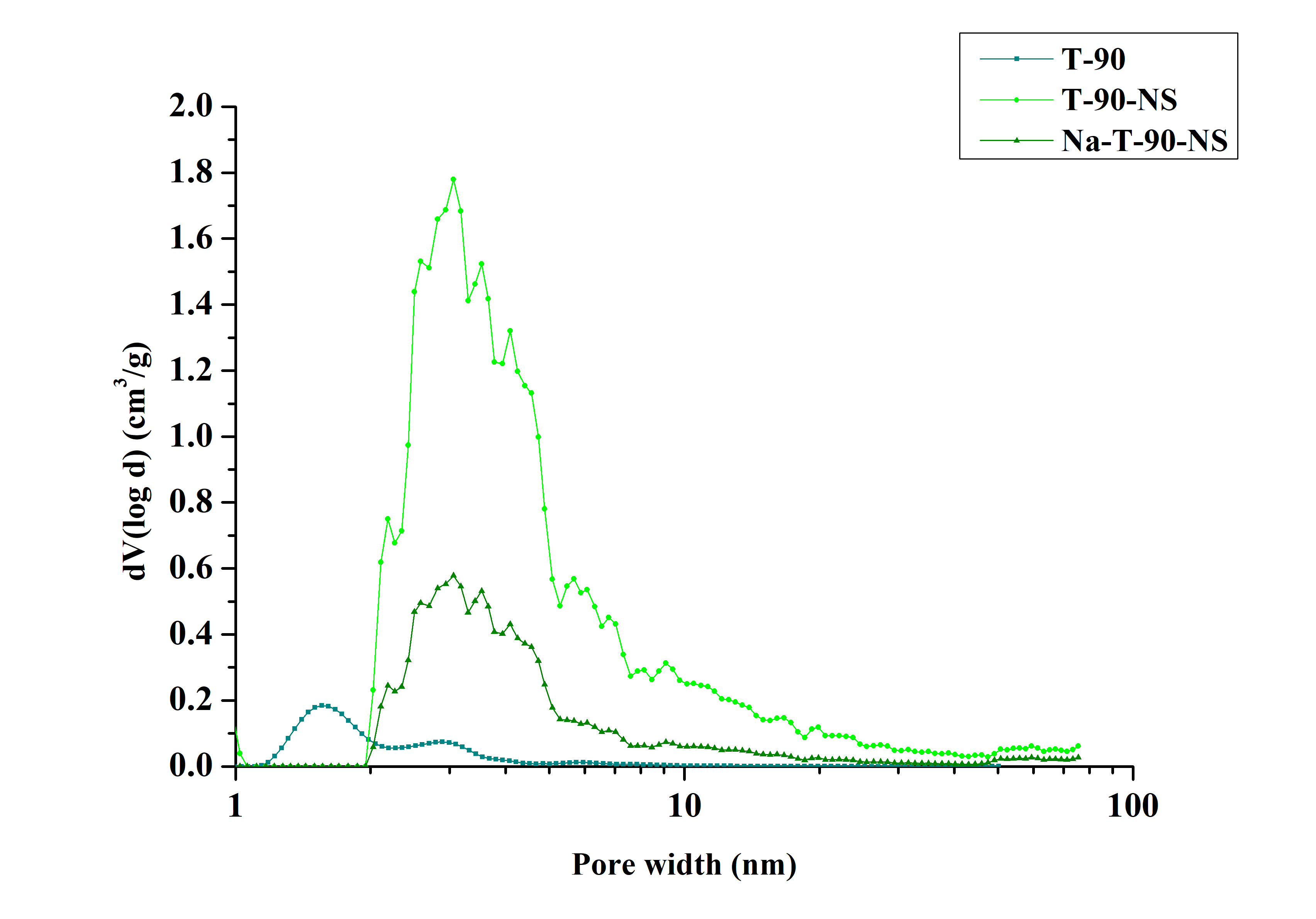


**Figure S9**: Pore size distribution of samples on variation of solvent


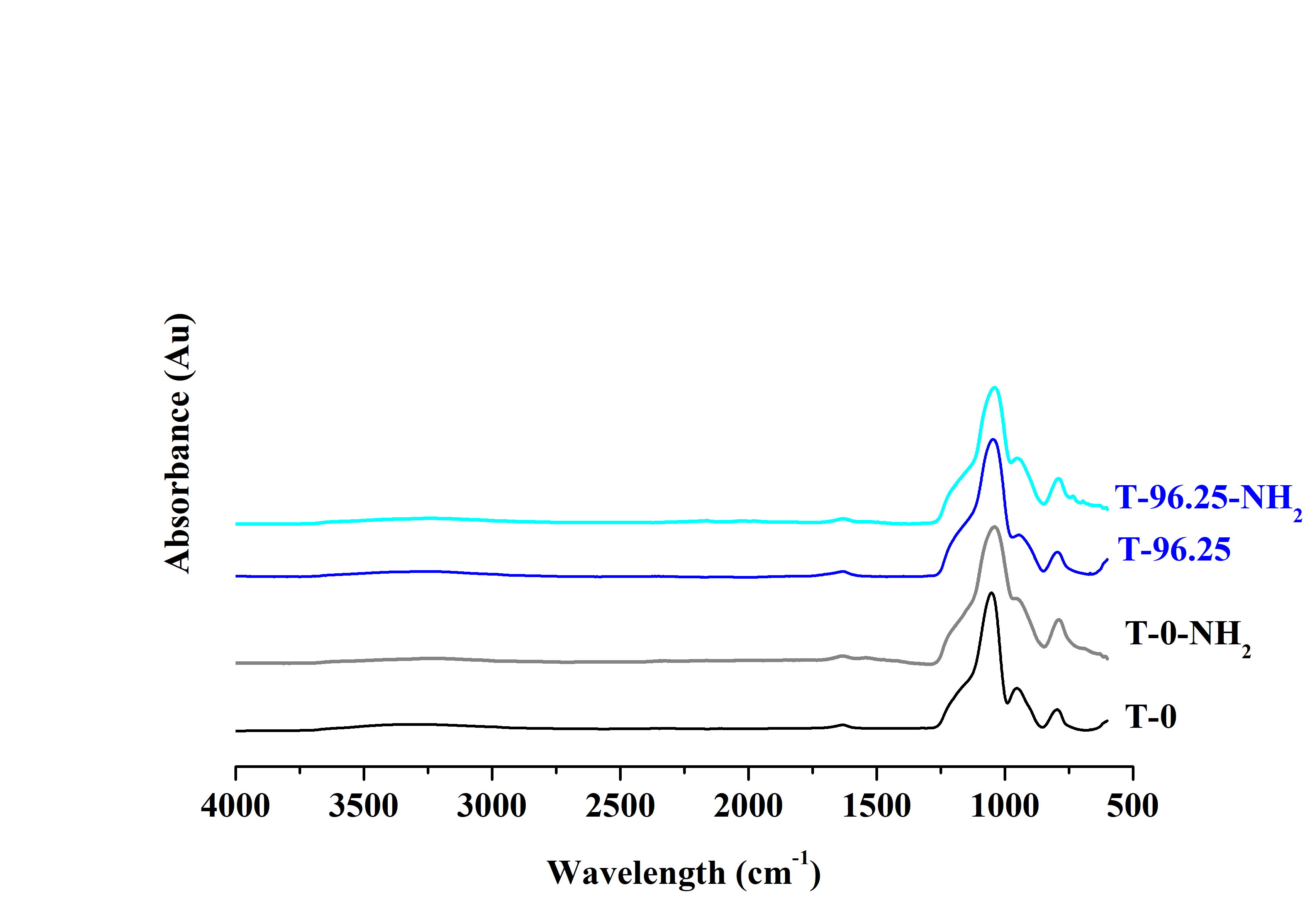


**Figure S10**: FTIR results of amine grafted samples


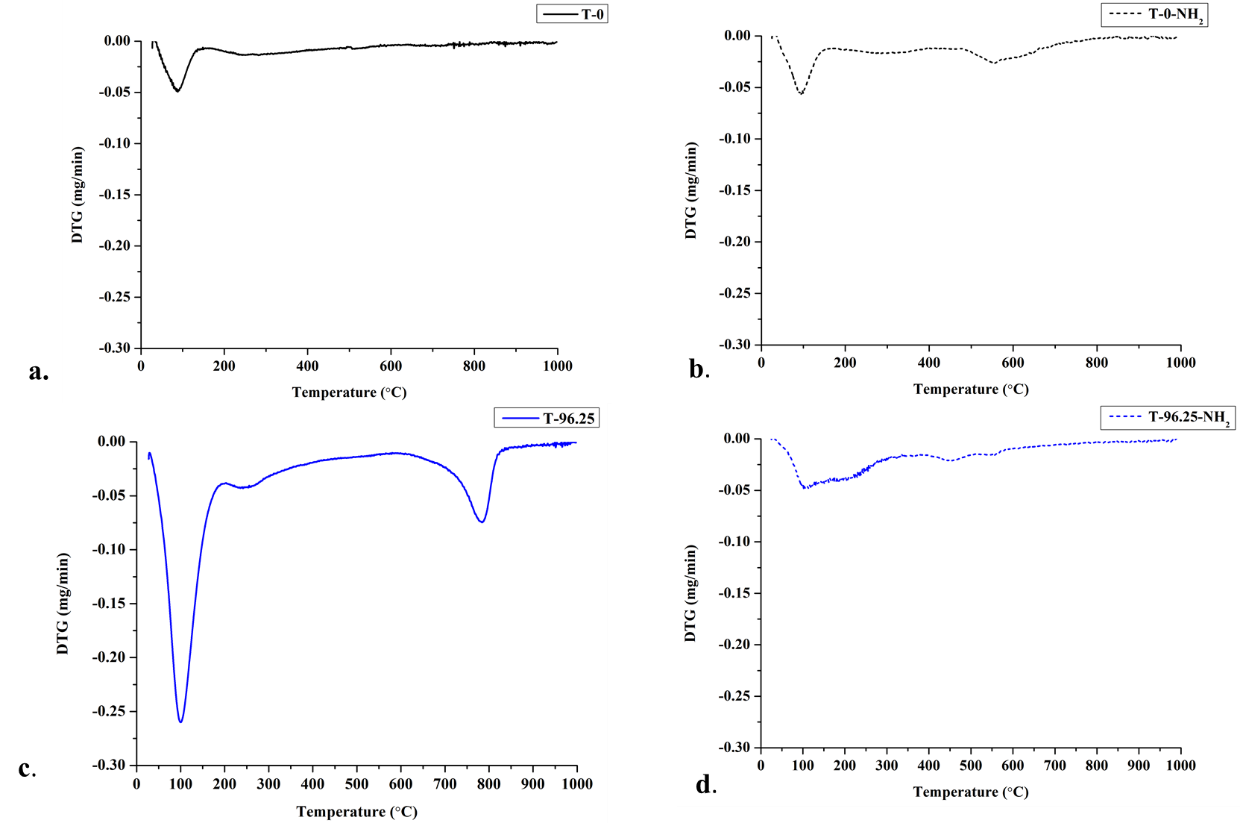


**Figure S11**: DTG profiles of T-0, T-0-NH_2_, T-96.25 and T-96.25-NH_2_

**Table S1** Parameters from universal isotherm model

|  | **T-0** | **T-0-NH_2_** | **T-97.25** | **T-96.25** | **T-96.25-NH_2_** | **T-95** | **T-95-AlO(OH)** | **T-90** | **T-90-NS** | **Na-T-90-NS** | **T-66.67** | **Faujasite** |
| --- | --- | --- | --- | --- | --- | --- | --- | --- | --- | --- | --- | --- |
| **E_1_ (+hfg)** | 11254.74 | 16775.74 | 10143.7 | 11762.7 | 10003.74 | 12482.7 | 11844.74 | 12445.7 | 11844.7 | 12505.7 | 10074.7 | 12195.74 |
| **E_2_ (+hfg)** | 8106.74 | 9329.74 | 7560.74 | 8352.74 | 7525.74 | 8674.74 | 8429.74 | 8647.74 | 8352.74 | 8625.74 | 7487.74 | 7371.74 |
| **E_3_ (+hfg)** | 6513.74 | 6884.74 | 6266.74 | 6584.74 | 6236.14 | 6708.74 | 6616.74 | 6662.74 | 6565.74 | 6694.74 | 6201.04 |  |
| **E_4_ (+hfg)** | 5662.24 | 5766.64 | 5562.74 | 5685.64 | 5541.74 | 5719.14 | 5692.74 | 5702.44 | 5679.54 | 5729.74 | 5537.04 |  |
| **α_1_** | 28.79 | 19.1 | 27.91 | 28.86 | 19.92 | 26.06 | 26.67 | 29.17 | 27.17 | 28.6 | 23.5 | 79.32 |
| **α_2_** | 32.25 | 36.08 | 32.5 | 33.61 | 31.87 | 35.03 | 34.85 | 34.28 | 33.29 | 33.04 | 33.51 | 20.68 |
| **α_3_** | 24.84 | 28.7 | 25.54 | 23.99 | 30.1 | 24.9 | 24.82 | 24.16 | 24.81 | 24.11 | 28.1 |  |
| **α4** | 14.12 | 16.12 | 14.05 | 13.54 | 18.11 | 14.12 | 13.66 | 12.46 | 14.73 | 14.25 | 14.89 |  |
| **m1** | 1390 | 3327 | 1042 | 1702 | 1066 | 1879 | 1743 | 1877 | 1807 | 1863 | 1180 | 1658 |
| **m2** | 665.5 | 1050 | 548.6 | 751.2 | 519.7 | 837.5 | 772.4 | 829.8 | 751.7 | 806.3 | 542.8 | 760.4 |
| **m_3_** | 338 | 449.5 | 271.4 | 348.7 | 269.3 | 386.6 | 359.2 | 379.9 | 352.3 | 382.6 | 254.9 |  |
| **m_4_** | 132.8 | 163.3 | 99.44 | 132.4 | 104.9 | 148.3 | 139.6 | 137.8 | 136.4 | 150.4 | 83.3 |  |
